# *Cordyceps and Beauveria* infections drive species-specific microbiome dysbiosis in the mosquito *Aedes aegypti*

**DOI:** 10.3389/fmicb.2026.1879658

**Published:** 2026-08-03

**Authors:** E. Everett, Haley M. Gore, Swaksha Kallepalli, Kristin R. Duffield, Lina Flor-Weiler, John Marino, Jose Luis Ramirez

**Affiliations:** 1 USDA-ARS, National Center for Agricultural Utilization Research, Crop BioProtection Research Unit, Peoria, IL, United States; 2 Department of Biology, Bradley University, Peoria, IL, United States

**Keywords:** entomopathogenic fungi, fungal biopesticides, microbial control, microbiome, mycopesticide

## Abstract

With the rising prevalence of vector-borne diseases and insecticide resistance in mosquitoes, alternative vector control strategies are urgently needed. Fungal entomopathogens offer a promising approach with a decreased likelihood of resistance development in mosquito populations. However, the mechanisms by which each fungus contributes to host mortality remain poorly understood, and the potential role of microbiome disruption as a secondary pathogenic mechanism has received limited attention. We evaluated the impact of four entomopathogenic fungal species (*Beauveria bassiana, Cordyceps javanica, C. cateniannulata*, and *C. amoenerosea*) on the microbiome of the yellow fever mosquito (*Aedes aegypti*) colonized with a defined, field-derived bacterial community. Whole-body bacterial communities were profiled using high throughput 16S rRNA amplicon sequencing, and community structure was assessed through alpha diversity metrics, beta diversity analysis, hierarchical clustering, and linear discriminant analysis effect size (LEfSe). All four fungal species successfully infected the mosquito; however, their effects on the mosquito microbiome were species-specific. *Cordyceps javanica* and *C. cateniannulata* reduced community evenness without significantly affecting species richness, a pattern consistent with a dominance-driven dysbiosis rather than broad bacterial loss. Infections by *C. amoenerosea* significantly increased total bacterial load and drove strong enrichment of the opportunistic genus *Pandoraea*, suggesting epithelial disruption or immune dysregulation as possible contributing factors. Beta diversity analysis indicated partial community-level restructuring across all fungal infections. *B. bassiana* showed a distinct genus-level compositional response, with enrichment of core symbiotic taxa and depletion of *Chryseobacterium* and *Kluyvera*, which was different from the Enterobacteriaceae-dominated shifts seen across infections with Cordyceps species. LEfSe analysis identified *Kluyvera* and *Burkholderia* as the strongest genus-level discriminators of infection state, suggesting potential utility as microbiome-based indicators of successful fungal colonization. These key findings were independently validated using EdgeR and batch-corrected MaAsLin2 analyses, with *Pandoraea* enrichment under *C. amoenerosea* and *Burkholderia* depletion under *C. cateniannulata* confirmed by both methods. Taken together, these results show that entomopathogenic fungi restructure the *Ae. aegypti* microbiome in a species-specific manner, inducing community destabilization and opportunistic bacterial enrichment that likely contribute to the detrimental effects of fungal infection. These results provide a mechanistic insights for the selection and development of fungal biopesticides for mosquito control.

## 1 Introduction

Vector-borne diseases represent one of the most significant threats to global public health, with a mortality burden of ~1.4 million deaths annually (Dorn et al., 2011). Among arthropod vectors, mosquitoes are of particular importance due to their role in transmitting pathogens that cause diseases such as yellow fever, Zika, West Nile, malaria and dengue (Duguma et al., 2020; Onen et al., 2023). Efforts to control these diseases have been hampered by the limited availability of vaccines or effective treatments (Scolari et al., 2019). Thus, management of vector-borne disease outbreaks still relies heavily on vector control, mainly through the use of synthetic chemical insecticides (Norris, 2004).

However, while synthetic chemical insecticides have historically been the primary tool for mosquito control, their indiscriminate use has led to the rapid development of resistance in all major mosquito vectors (Chaudhry et al., 2019; Meier et al., 2022; Perumal et al., 2024) and, also poses significant environmental risks impacting non-target organisms, including humans and other animals (Kumari et al., 2022; Zhou et al., 2025). These concerns highlight the urgent need for alternative mosquito control strategies. One promising approach is the use of microbial biopesticides, including bacterial and fungal entomopathogens, due to their specificity, reduced environmental impact, and lower potential for resistance development (Thakur et al., 2020; Salimi and Hamedi, 2021).

Currently, entomopathogenic fungi have been identified as a prospective agent of mosquito control, with the potential to kill the most important mosquito vectors (Scholte et al., 2007; Mnyone et al., 2009; Perumal et al., 2024). These fungi act by producing conidia that adhere to the mosquito cuticle in order to penetrate the mosquito exoskeleton and colonize the hemocoel. The mosquito is able to sense the fungal infection at the cuticle level via the action of pattern recognition receptors (PRRs) binding to pathogen or microbe associated molecular patterns (PAMPs or MAMPs) (Hughes, 2012; Hillyer, 2016; Rodgers et al., 2017; Ramirez et al., 2020) leading to the induction of several immune signaling pathways (Ramirez et al., 2019; Tikhe and Dimopoulos, 2021; Rhodes et al., 2025) and the production of antimicrobial peptides (Tawidian et al., 2019; Yan and Hillyer, 2019; Ramirez et al., 2023) that can suppress fungal proliferation. This response leads to a complex reciprocal interaction with direct and indirect effects of the host immune response on the microbiome (Ramirez et al., 2018; Gabrieli et al., 2021; Armitage et al., 2022; Zheng et al., 2023), pH alterations within the cuticle and hemolymph, and oxidative and melanization defenses that have an effect on the fungal pathogen and the host (Santiago-Álvarez et al., 2006; Nakhleh et al., 2017; Smith and Casadevall, 2019). The entomopathogenic fungi in turn secretes compounds (enzymes, elicitors, and secondary metabolites) aimed at suppressing the host immune response (Samuels R et al., 2016; Boucias et al., 2018; Qin et al., 2023). These fungal-derived compounds are thought to also be directed at inhibiting the host microbiome, as the fungi compete with resident microbial communities, which in turn can further modulate immune responses and impact host survival (Prompiboon et al., 2008; Boucias et al., 2018). Host death after fungal infection is preceded by the successful suppression of the host microbiota and immune response and is followed by emergence and sporulation on the host cadaver. This emergence is mediated by secondary metabolites produced by the fungi, such as oosporein, which act after host death to reduce competition by the microbiota before sporulation (Nielsen and Hajek, 2006; Fan et al., 2017).

The mosquito microbiome has gained attention for its vital role in host physiology, including effects on larval development, immune function, fecundity, and traits related to pathogen transmission (Hegde et al., 2018; Martinson and Strand, 2021). Mosquito-associated bacteria are influenced by biotic and abiotic factors including the mosquito genome, diet and geographical location (Muturi et al., 2018; Cansado-Utrilla et al., 2021). The host microbiota also plays a crucial role in mosquito reproduction and development, influencing key processes such as blood meal digestion, reproduction (Coon et al., 2016; Guégan et al., 2018) and successful larval pupation (Wang et al., 2018). During development, certain gut bacteria residents produce a hypoxia signal which promotes the transcription and initiation of processes needed for mosquito growth and development (Valzania et al., 2018). Furthermore, previous work on axenic (germ-free) mosquitoes has demonstrated significant physiological impairments, including altered gene expression related to nutrient absorption, metabolism, and stress response, alongside developmental failure, thereby highlighting the crucial physiological importance of the mosquito microbiome (Vogel et al., 2017).

Dysbiosis of the mosquito microbiota has been linked to altered vector competence, affecting arboviral transmission dynamics (Ramirez et al., 2012; Hegde et al., 2015; Gómez et al., 2022). Additionally, symbiotic bacteria such as *Wolbachia* can interfere with pathogen replication and influence vectorial competence (Johnson, 2015; Dacey and Chain, 2020). A deeper understanding of how these fungal infections impact the mosquito microbiome is critical for optimizing fungal virulence and enhancing biocontrol strategies. Identifying microbial changes in response to fungal infection can reveal key bacterial taxa that may play a role in host susceptibility and infection dynamics. Here, we define “dysbiosis” as infection-associated shifts in community evenness and composition relative to the inoculated community. However, mosquito microbiomes are inherently variable, and the concept of a stable “eubiotic” state in these systems is often contested (Hooks and O'Malley, 2017; Muturi et al., 2017).

While Wei et al. (2017) demonstrated that entomopathogenic fungi interact with the gut microbiota to accelerate mortality in *Anopheles gambiae*, that study focused on a single fungal species and the outcome of mortality rather than the specificity of microbiome restructuring. The present study extends this framework to *Aedes aegypti*, an important vector with different physiology and vectorial capacity. We also employ four distinct fungal species that for the first time allows direct within-study comparison of how different entomopathogenic fungal species differentially reshape the mosquito microbiome. This comparative analysis is valuable for guiding the selection of fungal biocontrol agents based not only on killing efficiency but on their specific effects on the mosquito microbiome, providing comprehensive insights into diverse host-pathogen interactions. Our results demonstrate that fungal infection significantly impacts microbiome composition and diversity in a strain-dependent manner, offering new insights into host-pathogen-microbiome interactions and guiding the development of future mosquito biocontrol strategies.

## 2 Materials and methods

### 2.1 Mosquito rearing

*Aedes aegypti* mosquitoes (Rockefeller strain) were reared under a 12 h light/dark cycle at 28°C and 70–80% relative humidity according to standard rearing conditions. Eggs were produced post blood feeding of adult females using an artificial membrane feeding system and bovine blood (HemoStat Laboratories Inc., Dixon, California, U.S.A.). Larvae were provided a 2:1:1 diet of rabbit food:liver powder:fish flakes and adults were maintained with a 10% sucrose solution in sterile cotton. Once emerged, adult mosquitoes were initially fed a 5% sterile sucrose solution supplemented with a defined bacteria set previously isolated from field-caught mosquitoes. This solution thereby mimicked the normal gut microbiota found in natural settings. The bacterial set consisted of seven taxa: *Pandoraea sputorum, Strenotrophomonas spp, Leclercia spp, Burkholderia spp, Pseudomonas spp*, and two strains of *Serratia marcescens*. Bacteria were incubated overnight at 27.4°C, 186 RPM, and concentrations were determined using a hemocytometer. A 5% sterile sucrose solution was used to adjust concentrations to 1 x 10^8^ cells/mL and provided to mosquitoes *ad libitum* via sterile cotton. Mosquitoes were maintained on this diet for 5 days until subsequent exposure to fungal pathogens (see below). This experimental design follows the synthetic community (SynCom) approach, where a defined assemblage of field-derived bacteria is used to colonize germ-free or conventionally reared hosts under controlled conditions (Coon et al., 2014; Sommer et al., 2015; Gould et al., 2018). This method offers significant advantages, including enhanced experimental reproducibility and the ability to attribute observed phenotypes to specific bacterial consortia. Moreover, SynCom approach allows for the incorporation of ecologically relevant taxa directly isolated from the target host population (Valzania et al., 2018).

### 2.2 Fungal infection assays

Four entomopathogenic fungal species were used for infection in this study: *Beauveria bassiana* (MBC 076), *Cordyceps javanica* (MBC 439), *Cordyceps cateniannulata* (MBC 6241), and *Cordyceps amoenerosea* (MBC 987). Fungi were cultured on Sabouraud dextrose agar and yeast extract (SDAY) medium and incubated at 20–25°C for 14 days. Spores were harvested the day of infection into 1 mL of soybean oil and vortexed for 10 min before being filtered through cheesecloth. Conidial concentrations were determined using a hemocytometer and adjusted to 1 x 10^8^ conidia/mL for *C. javanica, C. cateniannulata*, and *C. amoenerosea*, and 1 x 10^7^ conidia/mL for *B. bassiana*. The lower concentration used for *B. bassiana* was based on prior work with these strains and it was necessary to prevent early mortality and ensure sample collection at day 5 post-infection. Female mosquitoes were cold anesthetized 5d post emergence and 50.6 nL of the conidial oil suspension was topically deposited to the ventral surface of the coxal region using a Nanoject III micropipette (Drummond Scientific, Philadelphia, PA, USA). Control mosquitoes received 50.6 nL of soybean oil alone, following the same protocol. Two independent experiments were conducted, with 30 mosquitoes infected per treatment group in each experiment. From each experiment, 10 mosquitoes were individually collected per treatment group, yielding a total of 20 mosquito samples per treatment for downstream sequencing analysis. This number (*n* = 10 per experiment, 20 total per treatment) was selected taking into account mosquito mortality and the cost associated with sequencing. Fresh conidial oil suspensions were prepared independently for each experiment. Following infection, mosquitoes were maintained under the standard rearing conditions described above and provided a 10% sucrose solution.

### 2.3 DNA extractions and 16S rRNA amplicon sequencing

Mosquitoes were CO_2_ anesthetized 5 days post-infection, collected and stored at −80°C until DNA extraction. Individual mosquitoes were homogenized in a lysis buffer using a 3.2 mm and 2.4 mm beads in a TissueLyser II (Qiagen, Germany). Whole body mosquito DNA was extracted using the DNeasy^®^ Blood and Tissue Kit (QIAGEN, Netherlands) following the manufacturer's protocol and DNA concentration was measured using the Qubit 4 Fluorometer (Fisher Scientific, USA). PCR amplification was done on the V3 and V4 regions of the 16S ribosomal RNA gene (16S rRNA), yielding an amplicon product of ~460 bp. Reactions were prepared using 2x KAPA Hifi Hotstart ReadyMix (KAPA Biosystems Willington, MA), 2.5 μL of template DNA, and 0.5 μL of each primer (Supplementary Table S1) and included negative controls. Thermocycling conditions consisted of an initial denaturation step (3 min, 94°C) followed by 25 cycles consisting of a denaturation step (30s, 95°C), annealing step (15s, 58°C), and extension step at (15s, 72°C), followed by a final extension step (5 min, 72°C). Library quantification was done using the Qubit 4 Fluorometer (Fisher Scientific, USA) and samples were pooled, normalizing to 4 nM. Libraries were denatured with 0.2M NaOH and then diluted to 8pM. PhiX of the same concentration was added to make up 15% of the final pool and loaded onto the MiSeq V3 reagent cartridge. Libraries were sequenced on Illumina MiSeq with the 600 cycle v3 kit (Illumina) and 300 cycles of paired end reads. Whole-body microbiome profiling is common in mosquito microbiome studies (Hegde et al., 2018; Noskov et al., 2021) and captures bacteria that translocate to the hemocoel during fungal infection, which is relevant to fungal-infections of mosquitoes.

### 2.4 Quantitative real-time PCR for bacterial and fungal load

To confirm successful colonization and assess its effect on the overall bacterial community, we quantified fungal load (via 18S rRNA) and total bacterial load (via 16S rRNA) across all treatment groups. Bacterial and fungal loads were determined using quantitative PCR (qPCR) using SYBR^®^ Green Reagents (Qiagen) with samples being run on a 384-well block in the QuantStudio^TM^ 6 Flex System. Targets for quantification included 16S rRNA for bacterial load and ITS for fungal load (Supplementary Table S1). qPCR mixtures were prepared, in duplicate, using 5 μL of SYBR Green dye, 0.3 μL of both forward and reverse primers, 3.4 μL of deionized water, and 1 μL of the template DNA. Runs included a non-template negative control to test for potential contamination. qPCR was performed using an initial melting cycle (20s, 95°C), followed by 40 cycles of denaturation (1s, 95°C) and annealing and extension (20s, 60°C). Melt curves were generated following the initial PCR with a denaturation step (15s, 95°C), a cooling step (1 min, 60°C), and a final heating at a rate of 0.05 °C/s (15s, 95°C). Expression of these genes was normalized using the ribosomal protein *Rsp17* (AAEL004175) as a reference gene (Ramirez et al., 2021), and relative expression was calculated using the ΔΔC_T_ method (Livak and Schmittgen, 2001). Rps17 was selected as a reference gene because of its demonstrated stability across immune-challenged *Ae. aegypti* (Ramirez et al., 2019, 2020, 2021). This primer also provides a mosquito-specific signal that normalizes for variation in DNA input across individual samples. This normalization yields relative rather than absolute microbial load estimates.

### 2.5 Sequencing data processing and analysis

Samples for both experiments were sequenced in the same MiSeq run. Raw paired-end reads were demultiplexed based on barcode primer-tags and processed using the DADA2 pipeline, which included quality trimming, filtering, denoising, merging of paired reads, and chimera removal. Taxonomy was assigned against the SILVA reference database (version 138.2) at a 97% taxonomy similarity threshold. ASVs classified as archaea, mitochondria, chloroplasts were removed prior to downstream analysis. Alpha diversity was evaluated using observed ASVs, the Shannon Diversity Index, and the Simpson Diversity Index within the MicrobiomeAnalyst platform.

Beta diversity at the ASV level was analyzed by Principal Coordinate Analysis (PCoA) based on the Bray-Curtis dissimilarity matrix, with group-level differences tested by pairwise PERMANOVA. To assess the contribution of experimental replicate (experiment 1 vs. experiment 2) to whole community composition, we conducted a sequential PERMANOVA (Type-I SS, with 999 permutations) with “experiment” as the first term followed by “treatment” in R using the vegan package (adonis2 function). To visualize compositional differences across treatment groups, Z-score normalization was applied to the 16 most abundant bacterial genera and a heatmap was generated using hierarchical clustering. A five-way Venn diagram was also constructed to assess the number of shared and unique ASVs among control and fungal-infected groups.

Linear discriminant analysis effect size (LEfSe) analysis was performed to identify differentially abundant ASVs and genera among groups. A Kruskal-Wallis alpha value of 0.05 and an LDA threshold score of 2.0 were used to select discriminative features. Graphical outputs were used to visualize LDA scores and relative abundances (Supplementary Figures S3, S4). To validate the heatmap and LEfSe results and to identify additional associations, we used two differential abundance analyses for each fungal treatment vs. uninfected control. First, a Multivariable Association with Linear Models (MaAsLin2) (Mallick et al., 2021) was run as the primary validation, with experimental replicate as a fixed-effect covariate to account for between experiment variation. This analysis was performed at the ASV level using TSS normalization, log transformation, and a linear model (min_prevalence−0.1, BH FDR correction). Any significant ASVs were subsequently mapped to the genus using SILVA 138.2 taxonomy (Supplementary Table S5). This approach was selected as the primary validation because it uses the same TSS normalization as the original heatmap and the relative abundance analyses are conducted at the ASV level (similar to LEfSe analysis). More importantly, it takes into account the experimental batch-effects. Second, EdgeR (Robinson et al., 2010) was run as a complementary analysis using the EdgeR package. ASVs were filtered prior to testing using “filterByExpr” using min.count = 10, min.total.count = 15, min.prop = 0.7 (Chen et al., 2016). EdgeR differential abundance analysis for each fungal treatment vs. uninfected controls using the TMM normalization was applied, dispersions were estimated with “estimateDisp()”, and pairwise comparisons were evaluated using the “exactTest” and a Benjamini-Hochberg FDR threshold of 0.05 was used. Because the two methods differ in normalization method and the level at which the bacteria are tested, they are not expected to produce identical results.

### 2.6 Statistical analysis

All statistical analyses and visualizations were conducted in R (version R 4.2.2) using packages phyloseq, vegan, ggplot2, pheatmap, and in GraphPad Prism 10. Statistical differences in bacterial and fungal load were evaluated via Kruskal-Wallis test followed by Dunn's correction for multiple comparisons. Alpha diversity across groups were evaluated using Kruskal-Wallis tests followed by pairwise Wilcoxon rank-sum tests, with false discovery rate (FDR) correction for multiple comparisons.

## 3 Results

### 3.1 Fungal colonization and total bacterial load following entomopathogenic fungal infection

**Figure 1:**
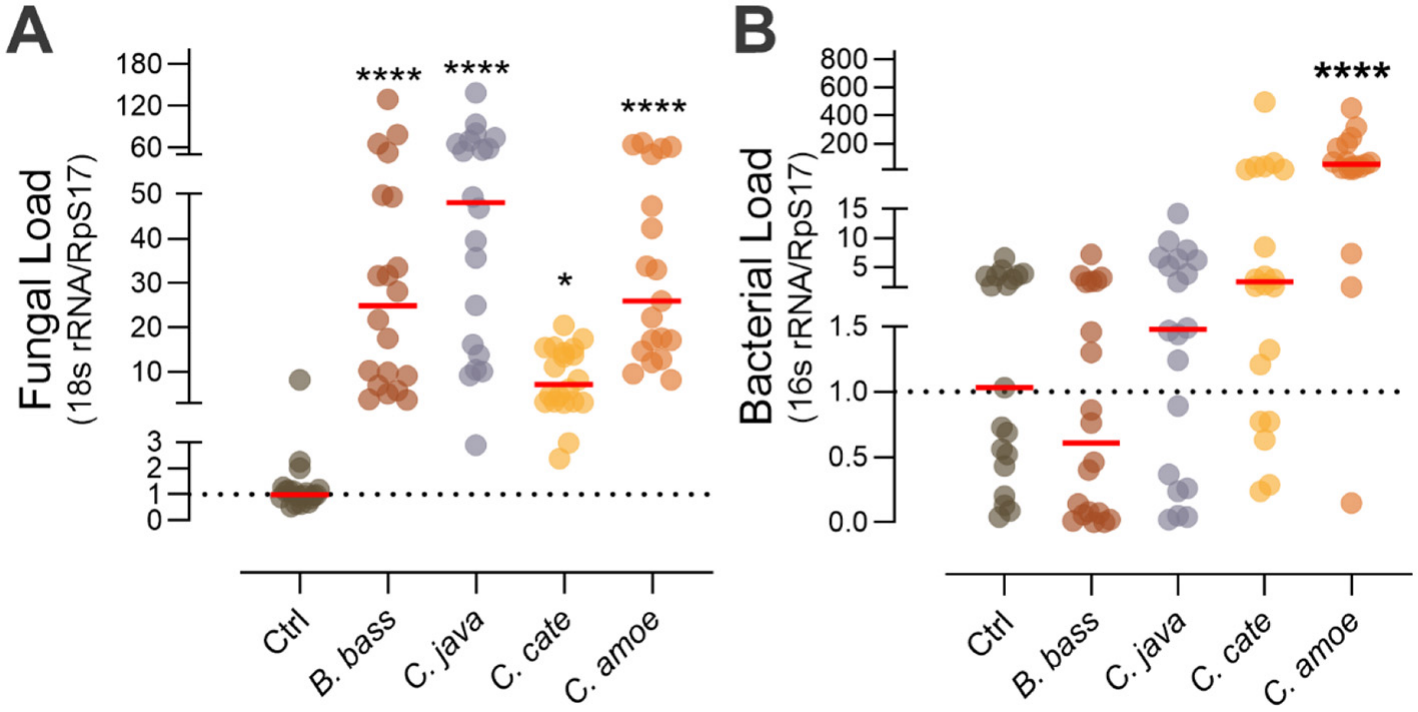
Fungal and bacterial load following infection with entomopathogenic fungi. Fungal load **(A)** and bacterial load **(B)** in control and fungal entomopathogen-infected mosquitoes. The red line represents the median, *n* = 20. Significant differences relative to uninfected controls determined by Kruskal-Wallis with Dunn's multiple comparison test. * *P* < 0.05, ****P < 0.0001. Ctrl, control; *B. bass, B. bassiana; C. java, C. javanica; C. cate, C. cateniannulata; C. amoe, C. amoenerosea*.

To evaluate whether entomopathogenic fungal infection influences the composition of the mosquito microbiome, *Ae. aegypti* mosquitoes were infected with one of four fungi tested: *C. cateniannulata, C. amoenerosea, B. bassiana*, or *C. javanica*. Our qPCR assays evaluating fungal load (via 18S rRNA) indicated that all four fungal species achieved statistically significant colonization relative to the uninfected controls (Figure 1A). *Cordyceps javanica* produced the highest median fungal load (~47-fold above controls), followed by *B. bassiana* and *C. amoenerosea* (both ~25 fold), and C. *cateniannulata* (~8-fold; *p* < 0.05). Notably *C. javanica* exhibited substantial inter-individual variability in fungal load, suggesting dose or host-dependent differences in infection. Quantification of bacterial load also showed shifts with fungal infection. In particular, mosquitoes infected with *C. amoenerosea* had significantly higher total bacterial loads than the control (Figure 1B). The remaining fungal treatments did not significantly alter total bacterial load, indicating that fungal colonization alone does not predict microbiome expansion.

### 3.2 Entomopathogenic fungal infections lead to genus-level compositional shifts in the mosquito microbiome

To discern the changes in microbiome diversity and composition that occurred under each treatment, we conducted amplicon-based metagenomics targeting the V3 and V4 region of the bacterial 16s rRNA gene from individual whole body mosquito DNA from each treatment group. After quality-filtering, there were a total of 3,285,282 reads with an average of 32,852 reads per sample. ASVs belonging to 4 phyla, 15 families, and 14 genera were identified (Supplementary Figures S1, S2).

**Figure 2:**
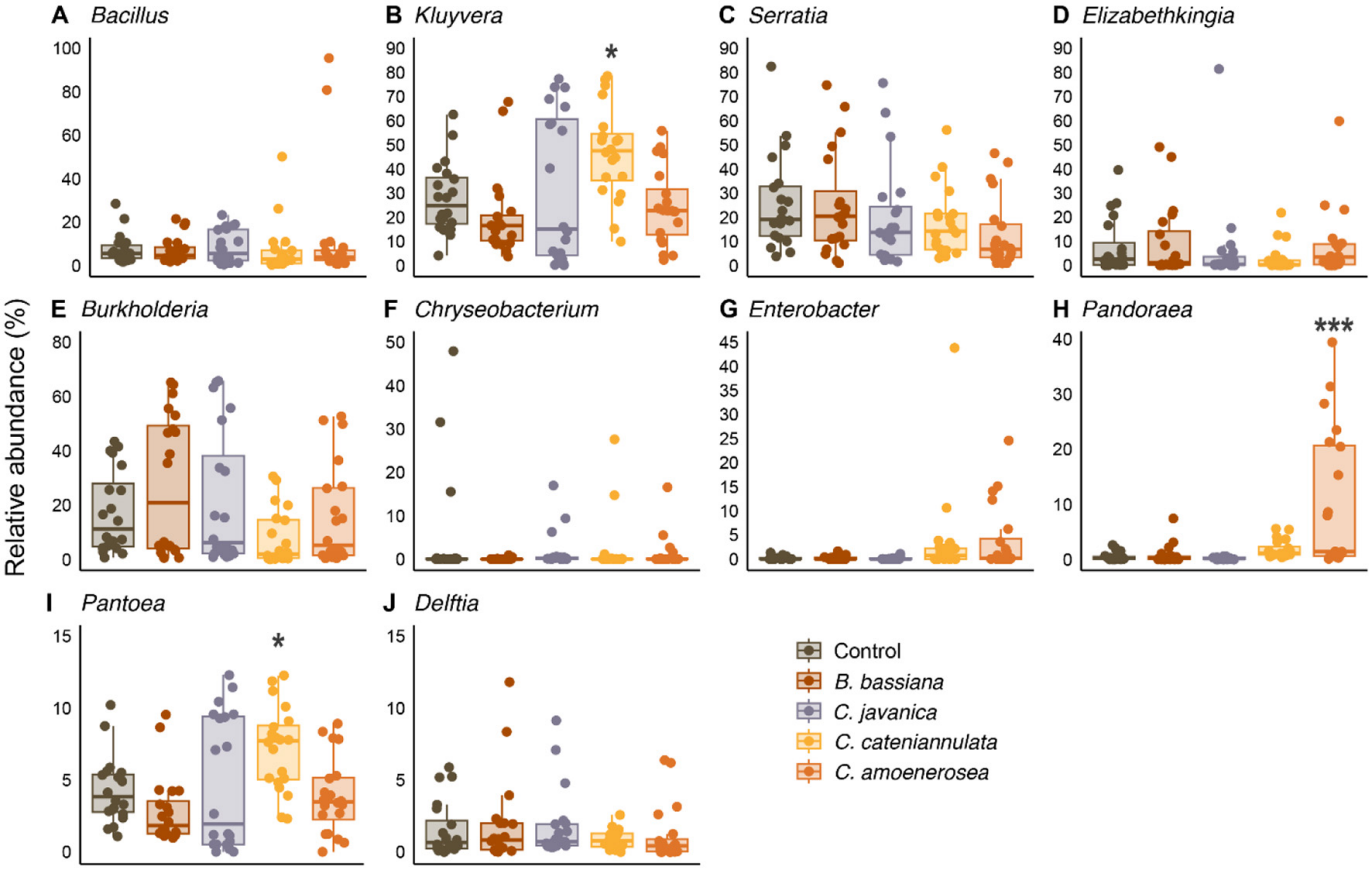
Genus-level relative abundance: Relative abundance of the 10 common bacterial genera in uninfected control mosquitoes and mosquitoes exposed to four different fungal entomopathogenic fungi. **(A)** *Bacillus* **(B)** *Kluyvera* **(C)** *Serratia* **(D)** *Elizabethkingia* **(E)** *Burkholderia* **(F)** *Chryseobacterium* **(G)** Enterobacter **(H)** Pandoraea **(I)** *Pantoea* and **(J)** *Delftia*. n=20, significant differences among treatment groups determined by Kruskal-Wallis test with Dunn's correction. *p < 0.05, ***p < 0.001.

To characterize the compositional changes at the genus level, we evaluated the relative abundances of the 10 most prevalent bacterial genera across treatment groups (Figure 2). *C. cateniannulata-*infected mosquitoes presented the highest relative abundance of both *Kluyvera* and *Pantoea* (Figures 2B, I) and *C. amoenerosea* had a significantly higher relative abundance of *Pandoraea* compared to all other treatment groups (Figure 2H).

### 3.3 Entomopathogenic fungal infection reduces microbiome diversity without altering richness

**Figure 3:**
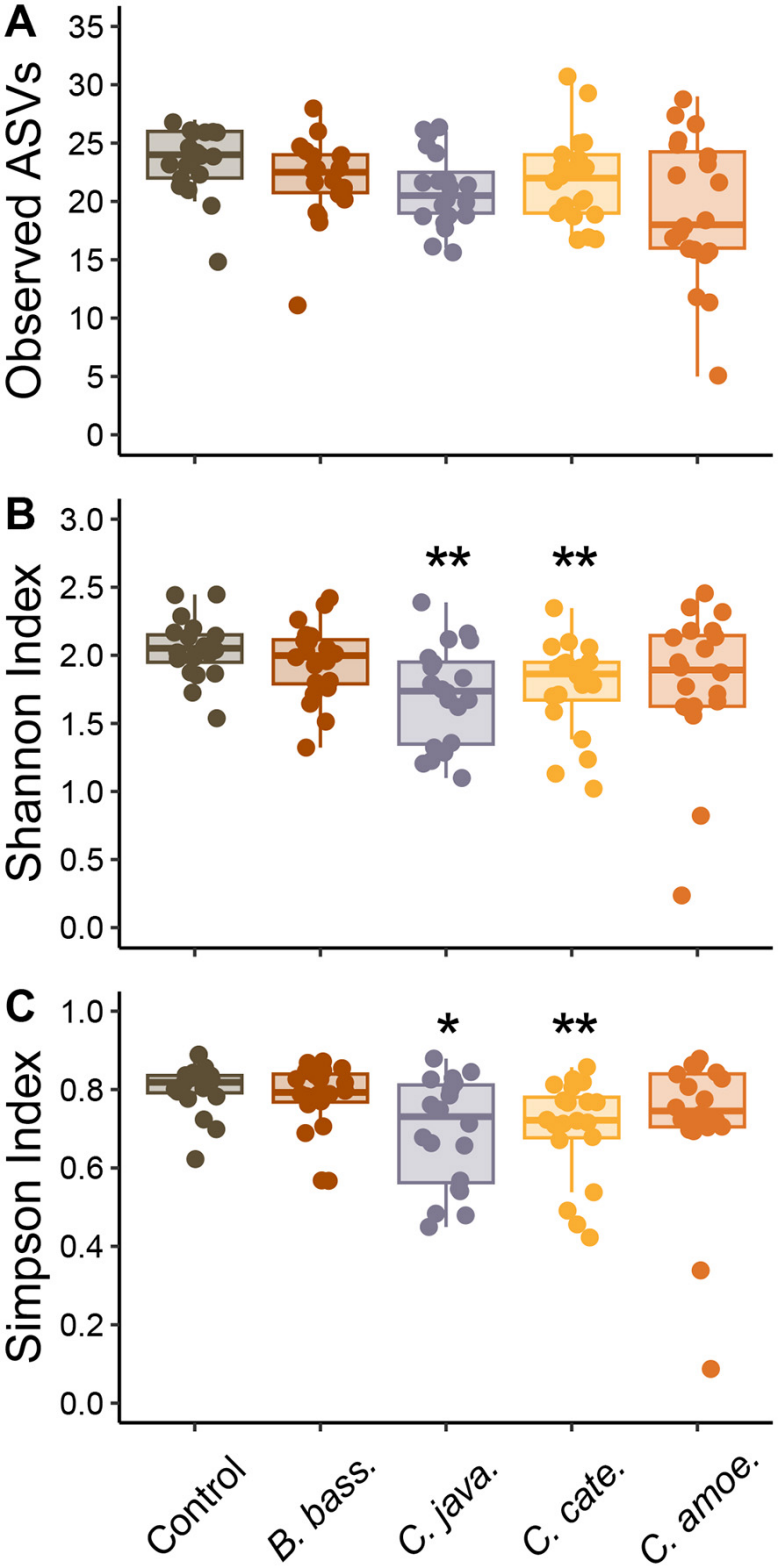
Alpha diversity metrics following infection with *B. bassiana, C. javanica, C. cateniannulata*, and *C. amoenerosea*. Metrics include the observed number of ASVs **(A)**, Shannon's index **(B)**, and Simpson's index **(C)**. *n* = 20, significant differences relative to uninfected controls determined by Wilcoxon rank-sum test with FDR correction *P < 0.05, **P < 0.01.

Alpha diversity was evaluated using observed ASVs as well as the Shannon and Simpson Indexes. The analysis of observed ASVs indicated no statistically significant differences detected (*p* < 0.05) and trends toward lower observed richness were noted in mosquitoes infected with *C. javanica* (strain 439) (*p* = 0.0845) and *C. amoenerosea* (strain 987) (*p* = 0.1391) relative to controls. A similar but weaker trend was also observed in mosquitoes infected with *C. cateniannulata* (strain 6241) (*p* = 0.1567) (Figure 3A and Supplementary Table S2). However, Shannon diversity indices were significantly lower in mosquitoes infected with *C. cateniannulata* (strain 6241) *and C. javanica* (strain 439) compared to uninfected controls (*p* = 0.0108 for both comparisons). No significant differences were detected between controls and mosquitoes infected with *B. bassiana* (strain 076) or *C. amoenerosea* (strain 987) or between the fungal-infected groups. Simpson diversity analysis revealed that mosquitoes infected with *C. cateniannulata* (strain 6241) and *C. javanica* (strain 439) exhibited significantly lower diversity compared to uninfected controls (*p* = 0.0104 and 0.0336, respectively). Additionally, Simpson diversity significantly differed between mosquitoes infected with *B. bassiana* (strain 076) and those infected with *C. cateniannulata* (strain 6241) (*p* = 0.0440). No significant differences were detected between other fungal-infected groups or between control and mosquitoes infected with *B. bassiana* (strain 076) or *C. amoenerosea* (strain 987) (Figures 3B–C and Supplementary Table S2).

### 3.4 Beta diversity reveals community restructuring following fungal infections

**Figure 4:**
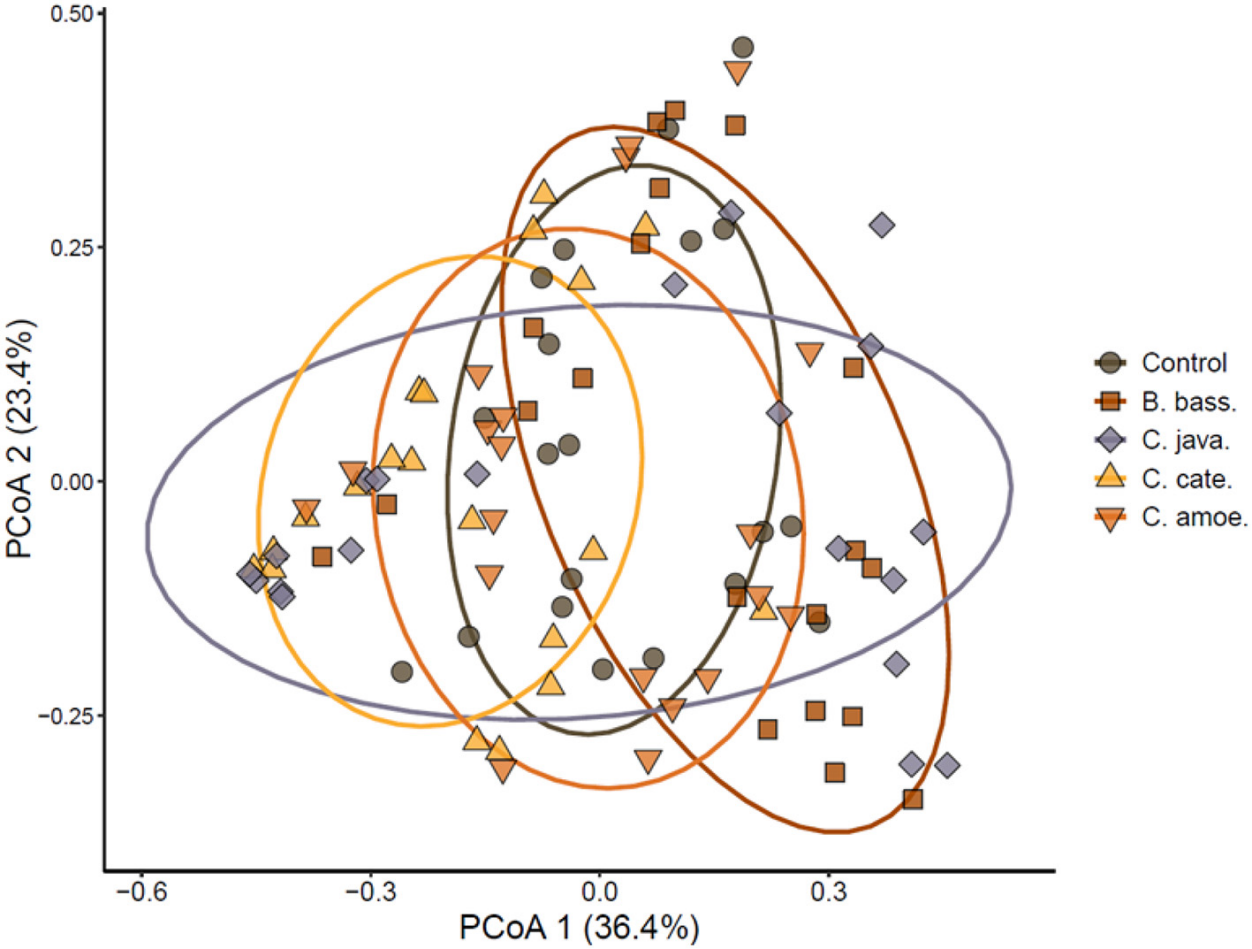
Beta diversity at the ASV level following infection with *B. bassiana, C. javanica, C. cateniannulata*, and *C. amoenerosea*. Principal coordinate analysis (PCoA) based on the Bray-Curtis dissimilarity matrix.

Beta diversity was analyzed to assess differences in microbiota composition between treatment groups using principal coordinate analysis (PCoA) based on the Bray-Curtis dissimilarity matrix. Beta diversity analysis at the ASV level indicated that the bacterial community composition differed significantly between uninfected controls and mosquitoes infected with *C. cateniannulata* (strain 6241) (*p* = 0.0067, *R*^2^ = 0.119) and between controls and mosquitoes infected with *C. amoenerosea* (strain 987) (*p* = 0.047) (Figure 4 and Supplementary Table S3). No significant differences were observed between controls and mosquitoes infected with *B. bassiana* (strain 076) or *C. javanica* (strain 439). In regard to differences observed between treatment groups, substantial differences were noted among fungal-infected groups, particularly between *B. bassiana* and *C. cateniannulata* (*p* = 0.005, *R*^2^ = 0.197), between *B. bassiana* and *C. amoenerosea* (*p* = 0.028, *R*^2^ = 0.069), between *C. cateniannulata* and *C. amoenerosea* (*p* = 0.005, *R*^2^ = 0.1) and between *C. cateniannulata* and *C. javanica* (*p* = 0.047, *R*^2^ = 0.076), indicating pathogen-specific impacts on microbiome composition.

To evaluate whether the two independent infection experiments contributed to the observed community composition differences, a sequential PERMANOVA was run with “experiment” entered as the first term followed by treatment (Type-I SS, with 999 permutations). The results indicated that “experiment” explained a significant portion of the total variance (*R*^2^ = 0.20, pseudo-*F* = 24.27, *p* < 0.001). This reflects the independent preparation of fungal suspensions and mosquito cohorts across runs. Furthermore, “treatment” explained a larger proportion of variance than “experiment” (*R*^2^ = 0.25, pseudo-*F* = 10.25, *p* < 0.001), and remained highly significant after accounting for batch effect. This indicates that the treatment effect reflects true biological differences in community composition rather than experimental run variation (Supplementary Table S3b). Pairwise comparisons that were adjusted for “experiment” indicated that three additional pairs were significantly different compared to the unadjusted model (*B. bassiana* vs. *C. javanica* (*p* = 0.002), *B. bassiana* vs. control (*p* = 0.006), and *C. javanica* vs. control (*p* = 0.001)). No previously significant comparison became non-significant after adjustment (Supplementary Table S3c). This indicates that the experimental batch effect was masking real treatment differences rather than creating artifacts. This also further strengthens the conclusion that each fungal species induces a distinct mosquito microbiome community during infection.

### 3.5 Hierarchical clustering identifies contrasting patterns of microbial genera disruption and replacement associated with fungal entomopathogenic infections

**Figure 5:**
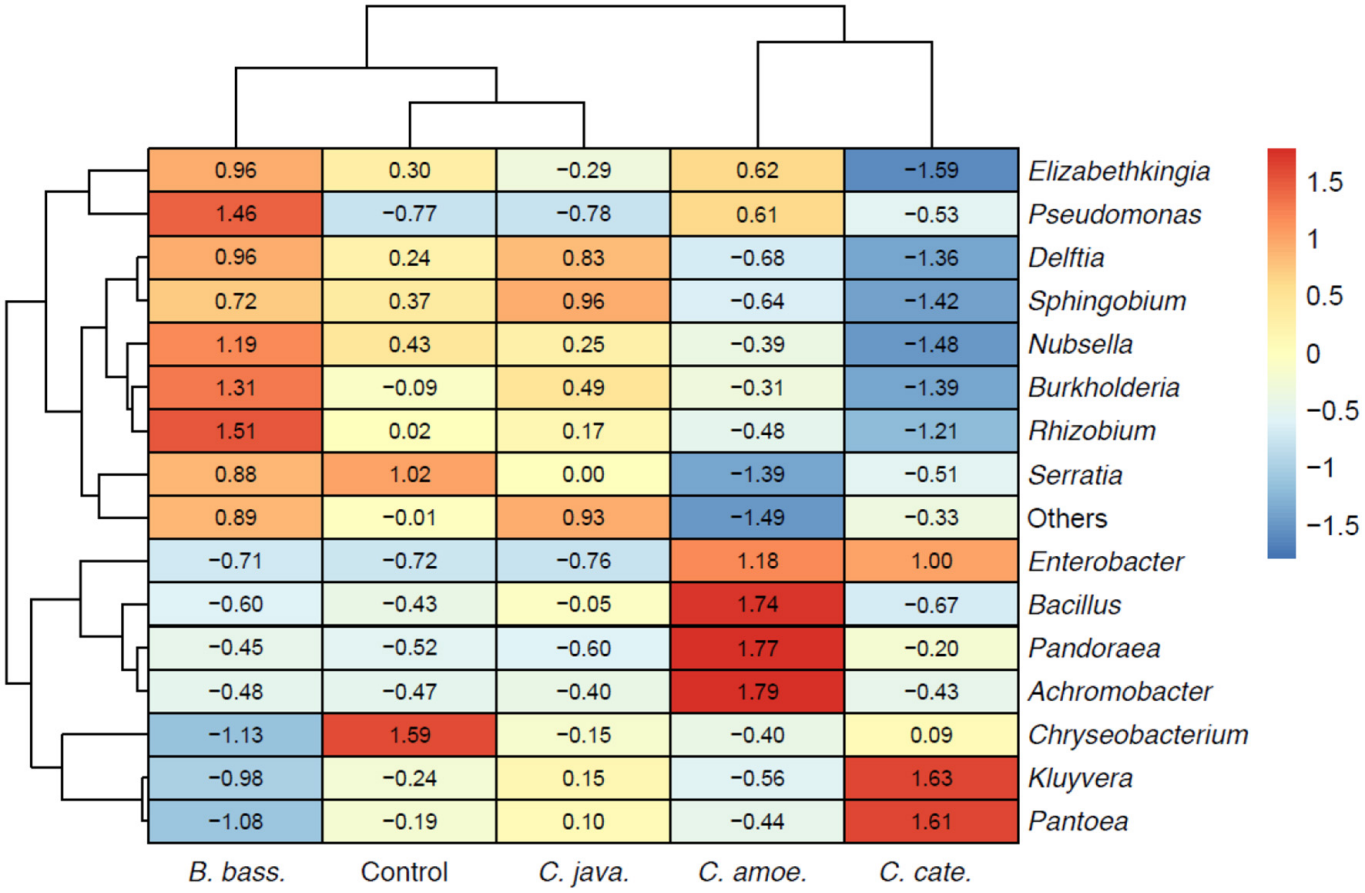
Differential impact of fungal entomopathogen infections on mosquito microbiome composition. Heatmap illustrating the Z-score normalized relative abundance of the 16 most abundant bacterial genera within the mosquito microbiome across infections with four different fungal entomopathogens. Each row represents a specific bacterial genus, and each column represents an experimental treatment: *B. bass*. (*B. bassiana* infection), Control (uninfected mosquitoes), *C. java*. (*C. javanica* infection), *C. amoe*. (*C. amoenerosea* infection), and *C. cate*. (*C. cateniannulata* infection). The Z-score in each cell quantifies the deviation of a genus's relative abundance from its mean across all samples, with positive values (red/orange) indicating enrichment and negative values (blue) indicating depletion. Both bacterial genera and treatment groups are hierarchically clustered based on similarity in their abundance profiles, as shown by the dendrograms. Two major treatment clusters are evident: *B. bassiana* (left cluster, enriched for *Pseudomonas, Nubsella, Burkholderia, Rhizobium*) and the three *Cordyceps* species (right cluster), with *C. amoenerosea* and *C. cateniannulata* showing the most pronounced shifts relative to controls.

Heatmap visualization was used to identify patterns of change in microbial composition and to identify taxa that differ between treatment groups (Figure 5). Hierarchical clustering at the genus level via z-score normalized abundances showed two major bacterial clusters that distinguished *B. bassiana* from the three *Cordyceps* species (Figure 5). Infection by *B. bassiana* resulted in enrichments of *Pseudomonas, Nubsella, Burkholderia*, and *Rhizobium*, with weaker enrichments observed within other genera. *B. bassiana* infections also led to a moderate reduction in *Chryseobacterium, Kluyvera* and *Pantoea* (Figure 5). The clustering data indicates that infections with *C. cateniannulata* and *C. amoenerosea* have a similar effect on the mosquito microbiome and the greatest changes in composition compared to the control. *C. amoenerosea* infection leads to a very strong enrichment of a specific cluster of bacteria: *Enterobacter, Bacillus, Pandoraea*, and *Achromobacter*. Simultaneously, *C. amoenerosea* strongly depletes many of the genera that were enriched by *B. bassiana* infection such as *Elizabethkingia, Serratia, Sphingobium*, etc. In turn, *C. cateniannulata* infection strongly enriches *Kluyvera* and *Pantoea*, and reduces many of the genera enriched in *B. bassiana* infection, such as *Elizabethkingia, Delftia, Sphingobium, Nubsella, Burkholderia*, and *Rhizobium*. Infections with *C. javanica* had the smallest effects on composition, clustering closer to the control group, with genera like *Delftia* and *Sphingobium* showing moderate enrichments with depletion of *Pseudomonas* and *Enterobacter*. Notable was the increase in the abundance of *Chryseobacterium* and *Serratia* in the uninfected control group. In contrast, *Chryseobacterium* was observed to be lower across all infection groups.

### 3.6 Venn diagram identifies shared and unique bacterial taxa between fungal entomopathogen infected groups

**Figure 6:**
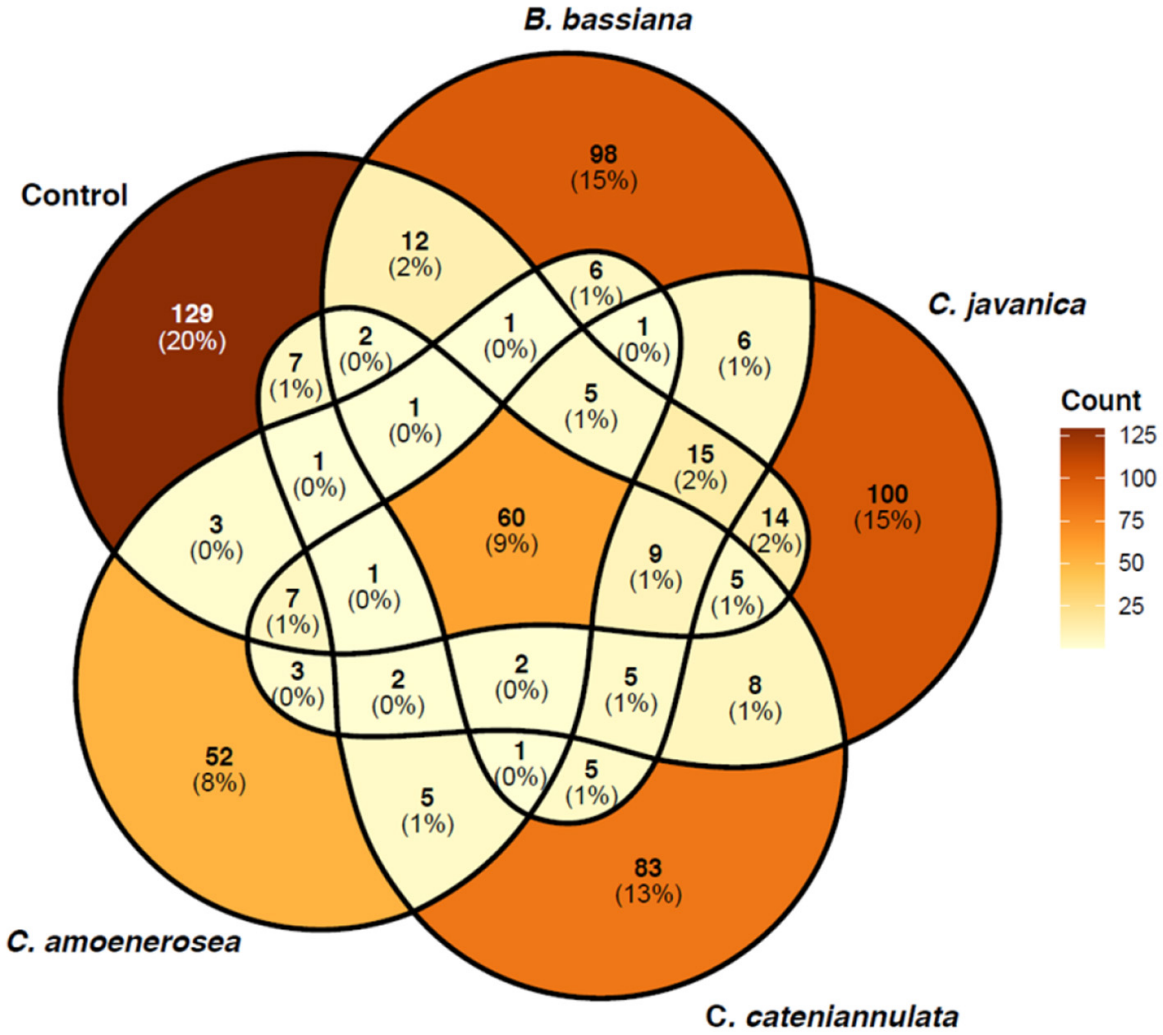
Shared and unique bacterial ASVs across fungal entomopathogen infection groups in mosquitoes. Venn diagram illustrates the distribution of unique and shared bacterial ASVs across control mosquitoes and those infected with four different fungal entomopathogens species: The color scale bar indicates the count of microbial ASVs within each region, ranging from 25 (light yellow) to 125 (dark brown). The diagram indicates a stable core microbiome alongside significant shifts in microbial community composition induced by different entomopathogenic fungal infections.

A Venn diagram was generated to view similarities in composition between treatment groups (Figure 6). This diagram highlighted shared and unique bacterial taxa between uninfected and fungus-infected mosquitoes. The Venn diagram reveals that fungal infections decreased the number of unique bacterial ASVs associated with the mosquito microbiome. Uninfected control mosquitoes harbored the highest number of unique ASVs (*n* = 129, 20% of total ASVs), whereas mosquitoes infected with *C. amoenerosea* exhibited the lowest number of unique ASVs (*n* = 52, 8%). Infections with *Cordyceps cateniannulata, B. bassiana*, and *C. javanica* also resulted in notable reductions in ASV richness compared to the control. Among all treatment groups, *B. bassiana* and *C. javanica* shared the most bacterial ASVs with the control with *C. amoenerosea* having the least shared ASVs with the control. A conserved core microbiome of 60 ASVs were found to be shared among all treatment groups and the control, with the control having 129 unique ASVs, *B. bassiana* having 98, *C. javanica* 100, *C. cateniannulata* 83, and *C. amoenerosea* 52 (Figure 6). Our results indicate that many taxa present in the control group are either lost or are undetectable after infection, while new taxa become dominant in the infected groups.

### 3.7 LEfSe analysis identifies discriminating taxa associated with entomopathogenic fungal infection

**Figure 7:**
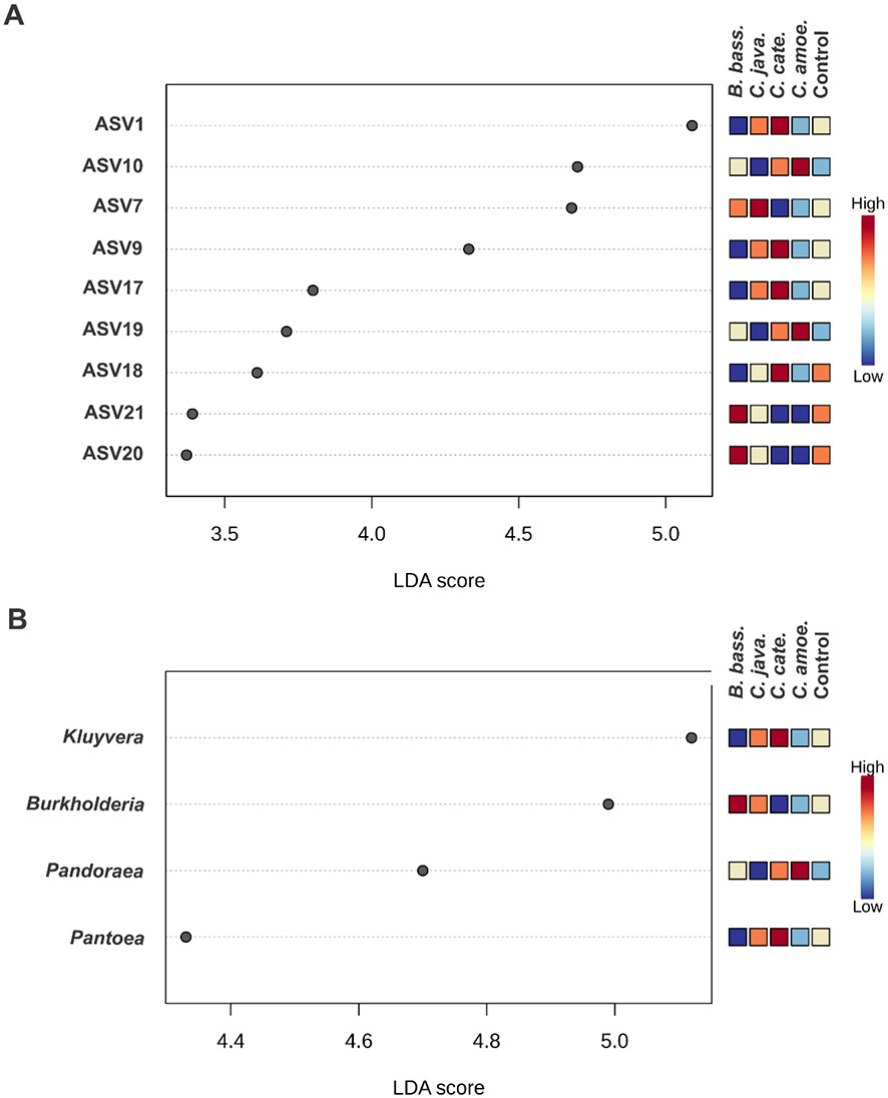
Linear discriminant analysis effect size (LEfSe) highlighting significant infection-associated discriminating taxa at the **(A)** ASV level and **(B)** genus level associated with fungal infection by each entomopathogen. Entomopathogenic fungi represented include *B. bassiana* (076), *C. javanica* (439), *C. cateniannulata* (6241), and *C. amoenerosea* (987).

Linear discriminant analysis effect size (LEfSe) was performed to identify ASVs and genera that strongly discriminate between each infection group. LEfSe analysis at the ASV level identified nine discriminating bacterial taxa significantly associated with different experimental groups with LDA scores ranging from approximately 3.3 to 5.1 (Figure 7A). Infections with *B. bassiana* favored ASV7, ASV21 and ASV20, while infections with *C. javanica* favored the increase of ASV7, and to a minor extent ASV9 and ASV17, while significantly decreasing ASV10 and ASV19 (Figure 7 and Supplementary Tables S4, S5). Infections with *C. cateniannulata* favored the increase of ASV1, ASV9, ASV17 and ASV18, while suppressing ASV7, ASV21, and ASV20. Infections with *C. amoenerosea* favored the increase of ASV10 and ASV19 while also suppressing ASV21 and ASV20. Control groups had equal representation with a slight increase of ASV18, ASV20 and ASV21 (See Supplementary Figure S3 for bar chart comparisons).

LEfSe analysis at the genus level identified four bacterial genera significantly associated with different treatments (Figure 7B). This analysis indicated that infections with *C. cateniannulata* favored the increase of the genus *Kluyvera* and *Pantoea* and a decrease of the genus *Burkholderia*; in particular, the genus *Kluyvera* showed the highest LDA score (~5.2), indicating a strong association (Figure 7B and Supplementary Table S6). Infections with *B. bassiana* favored the genus *Burkholderia* but suppressed the genus *Kluyvera* and *Pantoea*. A slight increase in the genera *Burkholderia* and *Kluyvera* as well as the suppression of *Pandoraea* were observed in mosquitoes infected with *C. javanica*. Infections with *C. amoenerosea* favored the increase of the genus *Pandoraea* but slightly suppressed genera *Kluyvera* and *Burkholderia* (Figure 7 and Supplementary Figure S4). These LefSe-identified discriminating taxa represent candidates based on within-dataset analysis and would require independent validation in additional mosquito cohorts or field-collected populations before being considered biomarkers of fungal infection.

### 3.8 MaAsLin2 and EdgeR analyses validate heatmap and LefSe findings and identify additional associations at the genus level

MaAsLin2 and EdgeR analysis confirmed the primary LefSe findings across all four fungal infections and identified additional associations at the genus level that were not detected by LEfSe (Supplementary Tables S7, S8 and Supplementary Figure S3). *Pandoraea* enrichment under *C. amoenerosea*, the strongest LEfSe discriminator for this species, was confirmed by both methods (MaAsLin2: Log2FC = +4.17, FDR = 4.22E-8; EdgeR: Log2FC = +4.85, FDR = 6.13E-9). *Burkholderia* depletion under *C. cateniannulata*, the second strongest LEfSe discriminator, was also confirmed by both methods (MaAsLin2: Log2FC = −2.74, FDR = 1.14E-5; EdgeR: Log2FC = −1.46, FDR = 4.81E-2). *Achromobacter* enrichment under *C. javanica* was confirmed by both methods (MaAsLin2: Log2FC = +2.03, FDR = 2.18E-2; EdgeR: Log2FC = +2.34, FDR = 4.67E-2), representing a new association not detected by LEfSe. *Enterobacter* enrichment was additionally confirmed by both methods under *C. amoenerosea* (MaAsLin2 FDR = 4.71E-5; EdgeR FDR = 2.81E-3) and *C. cateniannulata* infection (MaAsLin2 FDR = 5.95E-4; EdgeR FDR = 3.48E-2), also not among the LEfSe discriminators for either species.

Apart from these agreements across methods, MaAsLin2 recovered the two primary LEfSe enrichment signals for *C. cateniannulata*: *Kluyvera* (Log2FC = +1.09, FDR = 3.50E-3) and *Pantoea* (Log2FC = +0.47, FDR = 4.70E-2), which were not detected by EdgeR at the genus level, likely because these signals are specific to ASVs (which are diluted during genus-level aggregation). MaAsLin2 also identified depletion of *Burkholderia, Serratia, Sphingobium*, and *Nocardioides* under *C. amoenerosea* (all FDR ≤ 0.05). These agree with the heatmap showing a broad depletion of core microbiome members under this infection. EdgeR identified a broader depletion of core taxa under *C. cateniannulata*, including *Sphingobium, Elizabethkingia, Delftia, Serratia*, and *Nubsella* (all FDR ≤ 0.05) and the depletion of *Chryseobacterium* and *Kluyvera* under *B. bassiana* (FDR ≤ 2.40E-3). These are also in agreement with the heatmap. No genera reached significance under *B. bassiana* in MaAsLin2, reflecting the differences that exist at the ASV-level within the *Burkholderia*, with opposing directional shifts at the ASV level cancel during aggregation at the genus level. *Pseudomonas* enrichment under *C. amoenerosea* was identified by EdgeR (Log2FC = +3.28, FDR = 2.12E-3) and agrees in direction with a positive Z-score trend in the heatmap (+0.61), but did not reach significance in MaAsLin2, suggesting that this was a modest signal below the threshold for batch-corrected detection.

## 4 Discussion

Successful infection by entomopathogenic fungi involves suppression of both the mosquito's innate immune system and modulation of its resident microbiota (Butt et al., 2016; Wei et al., 2017; Ramirez et al., 2021). In this study, we characterized microbiome changes in *Ae. aegypti* following infection with four entomopathogenic fungi, revealing a dynamic and fungus-specific interaction with the mosquito microbiota. Our results highlight the profound impact of fungal infection on microbiome composition and structure.

One of the most important findings was the dissociation between fungal load colonization success and total bacterial load. All four entomopathogenic fungi achieved significant colonization of the mosquito body; however, only *C. amoenerosea*-infections led to a significant increase in total bacterial abundance. This suggests that bacteriome expansion is not a passive consequence of fungal infection intensity but rather reflects specific fungi-mosquito interactions. The genus most responsible for this increase was *Pandoraea*, a Betaproteobacterium that has been isolated from soil and insect guts, and some members are associated with opportunistic infection in immunocompromised hosts (Coenye et al., 2000; Yi et al., 2024). *Pandoraea'*s expansion under *C. amoenerosea's* infection could potentially indicate that this genus is promoting a state of immune dysregulation or may be disrupting the gut homeostasis that benefits bacterial proliferation. Similar patterns have been observed in other insect-fungal systems where immune suppression by entomopathogenic fungi facilitates bacterial expansion (Wei et al., 2017; Wang et al., 2023; Li et al., 2024b), which suggests that *C. amoenerosea* may mediate mosquito mortality through a dual mechanism of direct mycosis and bacterially-driven systemic stress. However, further experiments are necessary to link this phenotype to mosquito mortality.

Our findings also reveal that fungal infection affects the alpha diversity of the mosquito microbiome. The reductions in Shannon and Simpson indices without any corresponding changes in observed ASVs likely indicate that fungal infection induces community composition shifts driven by dominance rather than by bacterial elimination. This type of dysbiosis, in which community structure is shifted toward a small number of dominant opportunistic bacteria while other core taxa are relatively suppressed, leads to impairment of several host metabolic functions including disruption in immune homeostasis and larval development as well as increased susceptibility to secondary infections (Coon et al., 2016; Dickson et al., 2017; Li et al., 2024b). Thus, the significant reductions in evenness observed under *C. javanica* and *C. cateniannulata*-infections may translate to significant physiological costs to the mosquito apart from the direct effects of fungal infection. These findings are similar to previous reports of alpha diversity reductions following entomopathogenic fungal infections in insects (Noskov et al., 2021; Liu et al., 2023b; Mesquita et al., 2023) and suggest that dysbiosis these community shifts may contribute itself may contribute to accelerated mosquito mortality (Wei et al., 2017).

Although entomopathogenic fungi infect through the cuticle rather than the gut, the gut-associated bacterial community is nonetheless affected through systemic processes. The mechanism for these shifts is likely two fold: (1) the direct impact of fungal-derived enzymes, secondary metabolites and proteases that proliferate in the hemocoel and circulate systemically (Humber, 2008; Liu et al., 2023a; Zhang et al., 2024), potentially altering gut homeostasis indirectly; and (2) the collateral damage from the mosquito immune responses mounted to combat the fungal invasion (including oxidative stress, melanization, and antimicrobial peptide production) that indirectly suppresses commensal populations while leaving more resilient opportunists to fill the void (Krams et al., 2017; Boucias et al., 2018; McMillan and Adamo, 2020; Negroni et al., 2020). Experimental evidence from Wei et al. (2017) demonstrates that gut microbiota modulates mosquito susceptibility to fungal infection despite the infection initiating at the cuticle. This is further supported by Ramirez et al. (2018), who showed temporospatial immune modulation, including gut-associated immune responses, during cuticular fungal challenge in *Ae. aegypti*.

Beta diversity analysis further indicated that each fungal species induced unique shifts in microbial community composition across all treatment groups. For instance, mosquitoes infected with *C. cateniannulata* exhibited the most pronounced shift in overall community composition. This result indicates that fungal entomopathogenic species vary in their ability to restructure the mosquito microbiome, likely due to differences in virulence, systemic colonization, and metabolite production profiles (Bai et al., 2020; Peng et al., 2023). The magnitude of these differences has practical implications for how we conduct fungal biocontrol agent selection. Accounting for experimental replicate as a covariate in the PERMANOVA model reinforced these conclusions.

Heatmap clustering analysis indicated genus-level shifts in response to fungal infection. Specifically, it revealed a shared directional shift in community composition among *Cordyceps spp*. that is distinct from the *B. bassiana*-associated microbiome response. For instance, infections by *C. cateniannulata* resulted in an increase in the genera *Kluyvera* and *Pantoea* and a relative decline in genera that have been previously characterized as core components of the mosquito bacteriome, including *Elizabethkingia, Serratia, Nubsella, Delftia, Sphingobium, Burkholderia*, and *Rhizobium* (Kim and Lee, 2015; Bar-Shmuel et al., 2020; Li et al., 2020; Miyamoto et al., 2022; Gong et al., 2023; Chen et al., 2024). The loss of these taxa likely has critical functional consequences. *Elizabethkingia* is a genus commonly found in the microbiome of *Anopheles* and *Aedes* mosquitoes at all life stages and has been found to be associated with mosquito's physiology and has antiparasitic activity (Chen et al., 2024). Some *Delftia* species are known to produce the toxic alkaloid harmane which also has antiparasitic activity (Huang et al., 2023). Therefore, their decline could compromise the mosquito's resistance to parasites and opportunistic microbes, a shift that has the potential to promote mortality in the mosquito by enriching other opportunistic microbes residing in the gut. Two other important genera, *Nubsella* and *Burkholderia*, were also depleted. Some members of *Nubsella* have been found exclusively in healthy insects, and are thought to contribute to insect fitness and survival (Li et al., 2020), while *Burkholderia* is a well-characterized genus with many strains acting as insect symbionts with protective roles against entomopathogenic fungi and a putative role in digestion (Santos et al., 2004; Olivier-Espejel et al., 2011; Boissière et al., 2012). Thus, these losses could potentially lead to an impairment of immune competence and further enhance susceptibility to fungal infection. The decline of *Sphingobium* and *Rhizobium*, taxa involved in xenobiotic degradation and nitrogen fixation, respectively (Bar-Shmuel et al., 2020; Miyamoto et al., 2022), adds another layer of potential metabolic disruption. Given that uric acid production mitigates oxidative stress in blood-feeding insects (Weihrauch and O'Donnell, 2021), it is plausible that loss of nitrogen-fixing or xenobiotic-degrading bacteria may exacerbate ROS accumulation and accelerate mortality of fungus-infected mosquitoes. Alternatively, their decline may simply reflect sensitivity to the antimicrobial peptide production and oxidative defenses the mosquito mounts against the fungus. In any case, fungal entomopathogenic infections may not only perturb microbial diversity but may also displace functionally significant community members, potentially affecting metabolic processes such as detoxification and oxidative stress management.

The enrichment of *Kluyvera* and *Pantoea* specifically under *C. cateniannulata*, and not under the other fungal infections, further demonstrates *C. cateniannulata*' s ability to influence the mosquito microbiome. *Kluyvera* is a gram-negative Enterobacteriaceae found in insect gut microbiomes with some species been associated with degradation of foreign particles including polystyrene (Li et al., 2024a; Ndotono et al., 2024) and others are known to act as opportunistic entomopathogens causing mortality in certain insects (Ogodo, 2020). *Pantoea*, a bacterial genus frequently found in the *Aedes* mosquito, has been shown to influence vector competence through the secretion of anti-*Plasmodium* effector proteins (Ranasinghe et al., 2021). Expansion of *Pantoea* under *C. cateniannulata* infection most likely reflects opportunistic colonization of niches vacated by suppressed core taxa, plausibly due to host immune modulation, fungal metabolite production, and tissue damage that this species induces. Our LEfSe results, which identified both genera as strongly associated with *C. cateniannulata* treatment, support their potential utility as microbiome-based indicators of this specific fungal infection.

Infection by *C. amoenerosea* produced a community composition broadly similar to the *C. cateniannulata* group but with a distinct profile marked by relative increases in *Bacillus, Pandoraea*, and *Achromobacter* alongside a decrease of *Serratia*. The increase in *Bacillus* is of interest given that specific members of this genus have documented insecticidal activity (Toukabri et al., 2023; Deng et al., 2024) suggesting the possibility that its increase may contribute to mosquito mortality independently of the fungal infection. However, further research directly coupling fungal infection, microbiome profiling and survival is needed to link this microbiome disruption to mosquito mortality. Of significance was the statistically robust increase in bacterial load observed in *C. amoenerosea*-infected mosquitoes, which our analysis indicates *Pandoraea* as the primary driver of the bacterial load in this treatment group. While some *Pandoraea* species are opportunistic pathogens, others participate in the biodegradation of organic compounds and chemosynthetic processes (Kostygov et al., 2017); thus, its expansion may reflect a compensatory metabolic response to the nutrient and energetic demands of fighting off the fungal infection or may indicate an opportunistic proliferation in a disrupted microbiome. Some species of the genus *Achromobacter* are known to degrade many pesticides as a nutritional source (Pan et al., 2024), while others have insecticidal activity, suggesting that its increase could be either protective or harmful depending on which species is actually expanding. However, our genus-level data cannot determine which species are present. The reduction in *Serratia* under *C. amoenerosea* is also of significance. Given that certain *Serratia* strains have immunosuppressive qualities, with high cytotoxicity to granular cells and plasmatocytes (Kim et al., 2009; Toukabri et al., 2023), its decline may reflect active suppression by an upregulated mosquito immune response fighting off the fungal infection. Further experiments are needed to confirm the functional role of these bacteria.

Infection by *B. bassiana* was characterized by an increase in *Pseudomonas, Nubsella, Burkholderia*, and *Rhizobium* with a relative decrease in *Chryseobacterium, Kluyvera*, and *Pantoea*. The increase in *Pseudomonas* is particularly interesting given that certain members of this genus have been shown to provide nutrients and protection against entomopathogenic fungi, with *in vitro* studies identifying compounds produced by *Pseudomonas* that decrease the growth of entomopathogenic fungi (Saati-Santamaría et al., 2021). Its enrichment under *B. bassiana* infection could therefore represent a mosquito microbiome-level defense response with the recruitment or selective enrichment of bacteria capable of fighting the invading fungus. As previously mentioned, *Nubsella, Burkholderia*, and *Rhizobium* all play protective roles in insects such as increased fitness, protection against pathogens, and nitrogen fixation, respectively; thus, their retention and even enrichment under *B. bassiana* infection, in contrast to their suppression under *Cordyceps* species, further distinguishes this fungus from others. This differential retention of these taxa may also reflect differences in the secondary metabolite profiles these fungi deploy against the mosquito resident microbes.

Mosquitoes infected with *C. javanica* most closely resembled uninfected controls in overall microbial community composition, but one consistent feature across all fungal treatments (including *C. javanica*) was the reduction of *Chryseobacterium*. This genus was the most abundant taxon in the uninfected control group and was consistently reduced across all fungus-infected treatments, suggesting that it is playing a stabilizing role in the healthy mosquito microbiome. Its importance is further supported by previous studies demonstrating its high prevalence in the microbiomes of healthy insects (Shelomi et al., 2023; Akintola and Hwang, 2024) and its decline following fungal infections (Noskov et al., 2021). Members of the genus *Chryseobacterium* have been associated with immune homeostasis (Lee et al., 2016), and production of antimicrobial compounds that protect against opportunistic infections (Dahal et al., 2021). Thus, its consistent suppression across all fungal treatments may represent a shared early consequence of infection, removal of a key stabilizing taxon which consequently opens the door to secondary pathogens and opportunistic expansions described above. This makes *Chryseobacterium* a potential indicator for host microbiome health in the context of fungal-based microbial control strategies regardless of species.

Several of these genus-level associations were independently confirmed using two complementary differential abundance methods, MaAsLin2 (batch-corrected) and EdgeR. For example, MaAsLin2 and EdgeR results agree with the LEfSe analysis that *Pandoraea* was enriched under *C. amoenerosea* infection, indicating this genus as the primary indicator of *C. amoenerosea* infection. However, in some cases, associations were not detected by all three methods. For instance, MaAsLin2 indicated the enrichment of *Kluyvera* and *Pantoea* during *C. cateniannulata* infection, which also had the highest LEfSe LDA scores, while enrichment of these two genera was not detected by EdgeR, likely due to these signals belonging to specific ASVs that are diluted upon genus aggregation. On the other hand, EdgeR provided a wider coverage of core microbiome depletion under *C. cateniannulata* infection, which provided expanded information on the microbiome loss in addition to what MaAsLin2 and LEfSe detected. The three methods are therefore complementary rather than contradictory. The combined use of these two additional methods strengthens the conclusions drawn from the LEfSe and heatmap analysis.

While previous studies on mosquito-microbe interactions have focused mostly on single *Beauveria bassiana* strains, this approach overlooks the diverse pathogenic mechanisms of entomopathogenic fungi. Our study addresses this critical gap by investigating the effect of infection by four distinct *Beauveria* and *Cordyceps* strains on the microbiome of the yellow fever mosquito *Ae. aegypti* through infection bioassays and 16S rRNA metagenomic sequencing. The important changes in the core mosquito microbiome under the context of a fungal infections, described in the present study, likely have important consequences in mosquito physiology, immunity, and vector competence (Hegde et al., 2015; Song et al., 2018), making mosquitoes more susceptible to additional infections and/or accelerated mortality during fungal infections. However, further research is needed to elucidate interactions of observed taxa with entomopathogenic fungi and establish a causal link between the microbiome changes reported here and their potential contributions to mosquito mortality. From an applied point of view, these findings have direct relevance to the development and optimization of fungal biocontrol strategies. The fungal species-specific disruption of the mosquito microbiome documented in this study means that the choice of fungal agent should not just focus on killing efficiency, but also on its secondary microbiome-mediated effects on the mosquito host. The genera identified in this study, *Kluyvera* for *C. cateniannulata, Burkholderia* for *B. bassiana*, and *Pandoraea* for *C. amoenerosea*, could be useful as indicators of successful fungal colonization and microbiome disruption. This set of candidate bacterial discriminating taxa could aid in the development of novel strategies to enhance the efficacy of fungal entomopathogens in vector control programs.

Overall, our findings demonstrate that entomopathogenic fungal infections significantly reshape the mosquito microbiome. Infection by *C. cateniannulata* and *C. amoenerosea* showed the most pronounced disruptions. Although survival data were not collected in this study and it will be necessary to draw any correlation between microbiome disruption and host mortality, the findings reported in this study make a case for incorporating microbiome assessment as part of an entomopathogenic fungal biocontrol evaluation. Understanding how different fungal species reshape the mosquito microbiome adds a mechanistic dimension to biocontrol research and open new doors for refining the selection and application of fungal entomopathogens in integrated vector control programs.

## Data Availability

The original contributions presented in the study are included in the article/Supplementary material. Further inquiries can be directed to the corresponding author/s.
