# Supplementary material for: Cordyceps and Beauveria infections drive species-specific microbiome dysbiosis in the mosquito Aedes aegypti
Source: Front Microbiol. 2026 Aug 3;17:1879658. doi: 10.3389/fmicb.2026.1879658 (PMC13478364; doi:10.3389/fmicb.2026.1879658)
Supplement: Supplementary file 1 [file Data_Sheet_1.pdf]

Supplementary Tables

Supplementary Table S1. Bacterial primers for 16s rRNA PCR amplification, index PCR dual indices, qPCR primers, and Illumina sequencing adapters Nextera XT Index Kit (Illumina).

Target Group	Primer	Sequence (5'-3')	Reference
16S rRNA	16S Amplicon PCR Forward Primer	TCGTCGGCAGCGTCAGATGTGTATAAGAGACAG CCTACGGGNGGCWGCAG	Illumina
	16S Amplicon PCR Reverse Primer	GTCTCGTGGGCTCGGAGATGTGTATAAGAGACA GGACTACHVGGGTATCTAATCC	Illumina
Illumina Adapter	Forward Overhang	TCGTCGGCAGCGTCAGATGTGTATAAGAGACAG	Illumina
	Reverse Overhang	GTCTCGTGGGCTCGGAGATGTGTATAAGAGACA G	Illumina
Genfung	Genfung-F	AGATACCGTCGTAGTCTTAACCATAAACT	Bell et al. 2009
	Genfung-R	TTCAGCCTTGCGACCATACT	Bell et al. 2009
16s rRNA (qPCR)	16S rRNA-Fw	TCCTACGGGAGGCAGCAGT	Nadkarni et al. 2002
	16S rRNA-Rev	GGACTACCAGGGTATCTAATCCTGTT	Nadkarni et al. 2002
Rps17	Aed-rps17-F	TCCGTGGTATCTCCATCAAGCT	Joubert et al. 2016
	Aed-rps17-R	CACTTCCGGCACGTAGTTGTC	Joubert et al. 2016

Supplementary Table S2. Alpha diversity analyzed via Observed ASV index (A), Shannon's index (B), and Simpson's index (C). ** P< 0.01.

A.

**Pair**	**Statistic**	**P-value**	**FDR**
Control vs *B. bassiana*	267.0	0.069472	0.17368
*Control vs *C. javanica*	297.0	*0.008447	0.08447
*Control vs *C. cateniannulata*	273.5	*0.047017	0.15672
*Control vs *C. amoenerosea*	281.5	*0.027818	0.13909
B. bassiana vs *C. cateniannulata*	217.0	0.6535	0.6535
B. bassiana vs *C. javanica*	244.5	0.23154	0.33078
B. bassiana vs *C. amoenerosea*	252.0	0.16241	0.27068
C. cateniannulata vs *C. javanica*	224.0	0.52278	0.58086
C. cateniannulata vs *C. amoenerosea*	255.0	0.13913	0.27068
C. javanica vs *C. amoenerosea*	236.5	0.32856	0.4107

B.

**Pair**	**Statistic**	**P-value**	**FDR**
Control vs *B. bassiana*	241.0	0.27663	0.39518
**Control vs *C. javanica***	**315.0**	***0.001435**	**0.010828**
**Control vs *C. cateniannulata***	**311.0**	***0.0021655**	**0.010828**
Control vs *C. amoenerosea*	254.0	0.14928	0.29857
B. bassiana vs *C. cateniannulata*	272.0	0.0524	0.131
B. bassiana vs *C. javanica*	280.0	0.030411	0.10137
B. bassiana vs *C. amoenerosea*	226.0	0.49452	0.54947
C. cateniannulata vs *C. javanica*	219.0	0.62047	0.62047
C. cateniannulata vs *C. amoenerosea*	173.0	0.47769	0.54947
C. javanica vs *C. amoenerosea*	158.0	0.26483	0.39518

C.

**Pair**	**Statistic**	**P-value**	**FDR**
Control vs *B. bassiana*	226.0	0.49452	0.54947
**Control vs *C. javanica***	**299.0**	****0.006715**	**0.033575**
**Control vs *C. cateniannulata***	**318.0**	****0.0010412**	**0.010412**
Control vs *C. amoenerosea*	252.0	0.16534	0.33068
B. bassiana* vs *C. cateniannulata	**291.0**	**0.013194**	**0.043981**
B. bassiana vs *C. javanica*	280.0	0.030411	0.076028
B. bassiana vs *C. amoenerosea*	233.0	0.38341	0.47927
C. cateniannulata vs *C. javanica*	202.0	0.968	0.968
C. cateniannulata vs *C. amoenerosea*	155.0	0.2315	0.37833
C. javanica vs *C. amoenerosea*	158.0	0.26483	0.37833

Supplementary Table S3a. Beta Diversity analyzed via the Bray-Curtis Index at the ASV level generated using a pairwise PERMANOVA. ** P < 0.01. * P< 0.05.

Pair	F-value	R-squared	P-value	FDR
Control vs *B. bassiana*	1.786	0.044889	0.13	0.1625
Control vs *C. javanica*	0.93182	0.023935	0.409	0.409
**Control vs *C. cateniannulata***	**5.0407**	**0.11711**	****0.002**	**0.0066667**
**Control vs *C. amoenerosea***	**2.5308**	**0.062442**	***0.028**	**0.046667**
B. bassiana* vs *C. cateniannulata	**9.5625**	**0.20105**	****0.001**	**0.005**
B. bassiana vs *C. javanica*	1.6179	0.040837	0.163	0.18111
B. bassiana* vs *C. amoenerosea	**2.8371**	**0.069475**	***0.011**	**0.0275**
C. cateniannulata* vs *C. javanica	**3.1074**	**0.075593**	***0.024**	**0.046667**
C. cateniannulata* vs *C. amoenerosea	**4.1899**	**0.099311**	****0.001**	**0.005**
C. javanica vs *C. amoenerosea*	2.2647	0.056246	0.06	0.085714

Supplementary Table S3b. Sequential PERMANOVA (Type-I SS) assessing the contribution of experimental replicate (experiment) and treatment to bacterial community composition at the ASV level (Bray-Curtis dissimilarity, 999 permutations, n-100). Experiment was entered as the first term to partition its variance before treatment was tested. *** P < 0.001.

Source	df	SS	R^2	Pseudo-F	p-value
Experiment	1	3.41	0.199	24.27	< 0.001 ***
Treatment	4	4.31	0.251	10.25	< 0.001 ***
Residual	90	9.47	—	—	—
**Total**	99	17.2	—	—	—

Supplementary Table S3c. Pairwise PERMANOVA comparing bacterial community composition between treatment groups, unadjusted and after accounting for experimental replicate as a covariate (Bray-Curtis dissimilarity, 999 permutations, n=40 per pair). Light green shaded rows indicate comparisons that gained significance after adjustment. *P<0.05, **P<0.01, *** P < 0.001, ns = not significant.

Comparison	n	F (unadj.)	p (unadj.)		F (adj.)	p (adj.)
B. bassiana vs *C. amoenerosea*	40	2.98	0.012 *		7.83	0.001 ***
B. bassiana vs *C. cateniannulata*	40	9.39	0.001 ***		16.87	0.001 ***
B. bassiana vs *C. javanica*	40	1.73	0.150 ns		12.45	0.002 **
B. bassiana vs Control	40	1.75	0.125 ns		6.42	0.006 **
C. amoenerosea vs *C. cateniannulata*	40	4.07	0.003 **		8.48	0.001 ***
C. amoenerosea vs *C. javanica*	40	2.36	0.044 *		13.47	0.001 ***
C. amoenerosea vs Control	40	2.53	0.017 *		5.76	0.002 **
C. cateniannulata vs *C. javanica*	40	2.95	0.040 *		11.56	0.001 ***
C. cateniannulata vs Control	40	4.99	0.002 **		8.13	0.006 **
C. javanica vs Control	40	1.05	0.344 ns		13.91	0.001 ***

Supplementary Table S4. Linear Discriminant Analysis Effect Size (LEfSe) using the Kruskal-Wallis rank sum test. Features are ranked by their P values indicated by: **** P< 0.0001, *** P< 0.001, ** P < 0.01. * P< 0.05.

Name	P-value	FDR	076	439	6241	987	Control	LDAscore
**ASV10**	******1.1617E-8**	**4.1821E-7**	**8400.3**	**1909.2**	**18730.0**	**102030.0**	**5446.1**	**4.7**
**ASV7**	******1.2997E-6**	**2.3395E-5**	**76789.0**	**103280.0**	**8098.9**	**23252.0**	**45458.0**	**4.68**
**ASV17***	******6.6264E-6**	**7.9516E-5**	**1406.8**	**6270.1**	**13967.0**	**2291.0***	**4803.1**	**3.8**
**ASV18**	*****5.3018E-4**	**0.0047717**	**1828.8**	**5426.0**	**9887.5**	**4622.2**	**5928.5**	**3.61**
**ASV9**	****0.0018472**	**0.0133**	**28637.0**	**47548.0**	**71704.0**	**38947.0**	**42866.0**	**4.33**
**ASV1**	****0.002658**	**0.015948**	**200700.0**	**306790.0**	**445000.0**	**239150.0**	**267750.0**	**5.09**
**ASV20**	****0.0064091**	**0.032961**	**5405.9**	**2210.6**	**743.57**	**1004.8**	**2793.4**	**3.37**
**ASV21**	***0.011088**	**0.049898**	**5888.3**	**2049.8**	**1024.9**	**1145.5**	**2632.6**	**3.39**
ASV19	*0.013273	0.053091	522.51	160.77	9063.5	10350.0	341.64	3.71

Supplemental Table S5. Classification of bacterial ASVs to kingdom, phylum, class, order, family, and genus.

#Taxonomy	Kingdom	Phylum	Class	Order	Family	Genus
ASV1	Bacteria	Proteobacteria	Gammaproteobacteria	Enterobacterales	Enterobacteriaceae	Kluyvera
ASV10	Bacteria	Proteobacteria	Gammaproteobacteria	Burkholderiales	Burkholderiaceae	Pandoraea
ASV7	Bacteria	Proteobacteria	Gammaproteobacteria	Burkholderiales	Burkholderiaceae	Burkholderia-Caballeronia-Paraburkholderia
ASV9	Bacteria	Proteobacteria	Gammaproteobacteria	Enterobacterales	Enterobacteriaceae	NA
ASV17	Bacteria	Proteobacteria	Gammaproteobacteria	Enterobacterales	Enterobacteriaceae	Kluyvera
ASV19	Bacteria	Proteobacteria	Gammaproteobacteria	Enterobacterales	Enterobacteriaceae	Enterobacter
ASV18	Bacteria	Proteobacteria	Gammaproteobacteria	Enterobacterales	Enterobacteriaceae	NA
ASV21	Bacteria	Proteobacteria	Gammaproteobacteria	Burkholderiales	Burkholderiaceae	Burkholderia-Caballeronia-Paraburkholderia
ASV20	Bacteria	Proteobacteria	Gammaproteobacteria	Burkholderiales	Burkholderiaceae	Burkholderia-Caballeronia-Paraburkholderia

Supplemental Table S6. Highly significant taxa indicators associated with fungal infection. **** P< 0.0001, *** P< 0.001, ** P < 0.01. * P< 0.05.

Name	P-values	FDR	076	439	6241	987	Control	LDAscore
Pandoraea	****1.1617E-8	2.5557E-7	8400.3	1909.2	18730.0	102030.0	5446.1	4.7
Pantoea	**0.0018472	0.016947	28637.0	47548.0	71704.0	38947.0	42866.0	4.33
Kluyvera	**0.0023109	0.016947	203940.0	318490.0	468850.0	246060.0	278480.0	5.12
Burkholderia	*0.016795	0.092374	275840.0	216180.0	79160.0	157640.0	173890.0	4.99

Supplemental Table S7. Differentially abundant genera identified by MaAsLin2 in R with experimental replicate as a fixed-effect covariate (TSS normalization, log transformation, linear model, BH FDR correction (Mallick et al 2021)). Analysis was performed at the ASV level and significant ASVs were subsequently mapped to genus using SILVA 138.2 taxonomy (Supplementary Table S5). Log_2FC values for genera represented by multiple significant ASVs reflect the mean across those ASVs. FDR 0.05. *FDR ≤ 0.05, ** FDR ≤ 0.01, *** FDR ≤ 0.001.

Genus	Direction	Log_2FC	p-value	FDR
***B. bassiana* (strain 076) vs. Control**				
No significant genera after batch correction (FDR > 0.05).				
***C. amoenerosea* (strain 987) vs. Control**				
Pandoraea	**Enriched**	4.17	3.08E-09	4.22E-8 ***
Enterobacter	**Enriched**	2.61	4.06E-06	4.71E-5 ***
Burkholderia	**Depleted**	−2.60	1.94E-04	1.41E-3 **
Serratia	**Depleted**	−1.36	2.68E-03	1.36E-2 *
Sphingobium	**Depleted**	−1.92	2.45E-03	1.18E-2 *
Nocardioides	**Depleted**	−1.54	1.18E-02	3.44E-2 *
***C. cateniannulata* (strain 6241) vs. Control**				
Burkholderia	**Depleted**	−2.74	5.34E-09	1.14E-5 ***
Pandoraea	**Enriched**	2.99	9.29E-06	3.70E-5 ***
Enterobacter	**Enriched**	1.65	3.41E-05	5.95E-4 ***
Kluyvera	**Enriched**	1.09	7.95E-05	3.50E-3 **
Nocardioides	**Depleted**	−1.54	1.32E-02	3.44E-2 *
Pantoea	**Enriched**	0.47	1.86E-02	4.70E-2 *
***C. javanica* (strain 439) vs. Control**				
Burkholderia	**Depleted**	−3.88	6.14E-06	1.06E-4 ***
Achromobacter	**Enriched**	2.03	1.07E-02	2.18E-2 *
Methylobacterium	**Depleted**	−1.42	2.64E-03	1.75E-2 *

Supplemental Table S8. EdgeR differential abundance analysis at the genus level. Differentially abundant genera identified by EdgeR in R (TMM normalization, filterByExpr filtering, min.count=10, min.total.count=15, min.prop=0.7 (Chen et al 2016) for each fungal treatment vs uninfected controls. FDR ≤ 0.05 (Benjamini-Hochberg). Log_2FC positive = enriched in infected group; negative = depleted. Green rows =enriched, red rows = depleted. * FDR ≤ 0.05, ** FDR ≤ 0.01, *** FDR ≤ 0.001.

Genus	Direction	Log_2FC	p-value	FDR
***B. bassiana* (strain 076) vs. Control**				
Chryseobacterium	**Depleted**	−6.04	5.34E-07	1.57E-3 **
Enterobacteriaceae	**Depleted**	−1.51	5.34E-07	1.57E-3 **
Kluyvera	**Depleted**	−1.34	2.64E-04	2.40E-3 **
Pseudomonas	**Enriched**	3.68	2.61E-06	2.40E-3 **
***C. amoenerosea* (strain 987) vs. Control**				
Pandoraea	**Enriched**	4.85	3.02E-10	6.13E-9 ***
Enterobacter	**Enriched**	3.93	1.88E-04	2.81E-3 **
Pseudomonas	**Enriched**	3.28	1.41E-04	2.12E-3 **
Bacillus	**Enriched**	2.45	1.88E-04	2.81E-3 **
***C. cateniannulata* (strain 6241) vs. Control**				
Sphingobium	**Depleted**	−2.44	1.58E-04	1.58E-3 **
Elizabethkingia	**Depleted**	−2.23	3.83E-05	1.03E-2 *
Delftia	**Depleted**	−1.79	1.35E-04	2.52E-3 **
Nocardioides	**Depleted**	−1.87	6.43E-04	6.43E-3 **
Nubsella	**Depleted**	−1.80	2.38E-04	1.03E-2 *
Rhizobiaceae	**Depleted**	−1.42	7.91E-04	7.91E-3 **
Serratia	**Depleted**	−1.65	4.93E-04	4.93E-3 **
Pseudacidovorax	**Depleted**	−1.64	1.97E-03	1.97E-2 *
Burkholderia	**Depleted**	−1.46	4.81E-03	4.81E-2 *
Bacillus	**Depleted**	−1.21	2.83E-03	2.83E-2 *
Enterobacter	**Enriched**	3.57	1.87E-04	3.48E-2 *
***C. javanica* (strain 439) vs. Control**				
Achromobacter	**Enriched**	2.34	1.01E-03	4.67E-2 *

Supplementary Figures

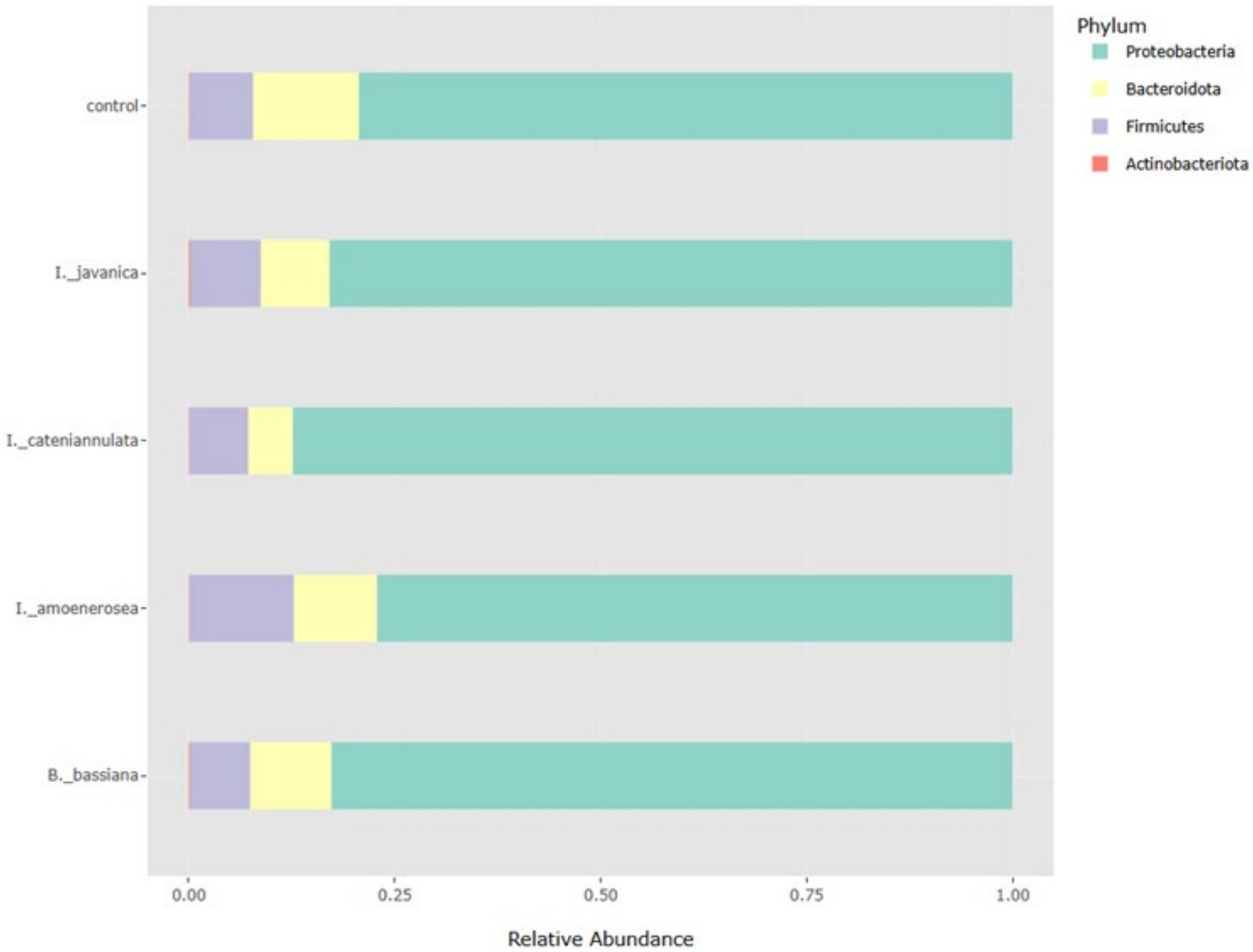

Supplementary Figure S1. Relative abundance of four bacterial phyla following fungal infection.

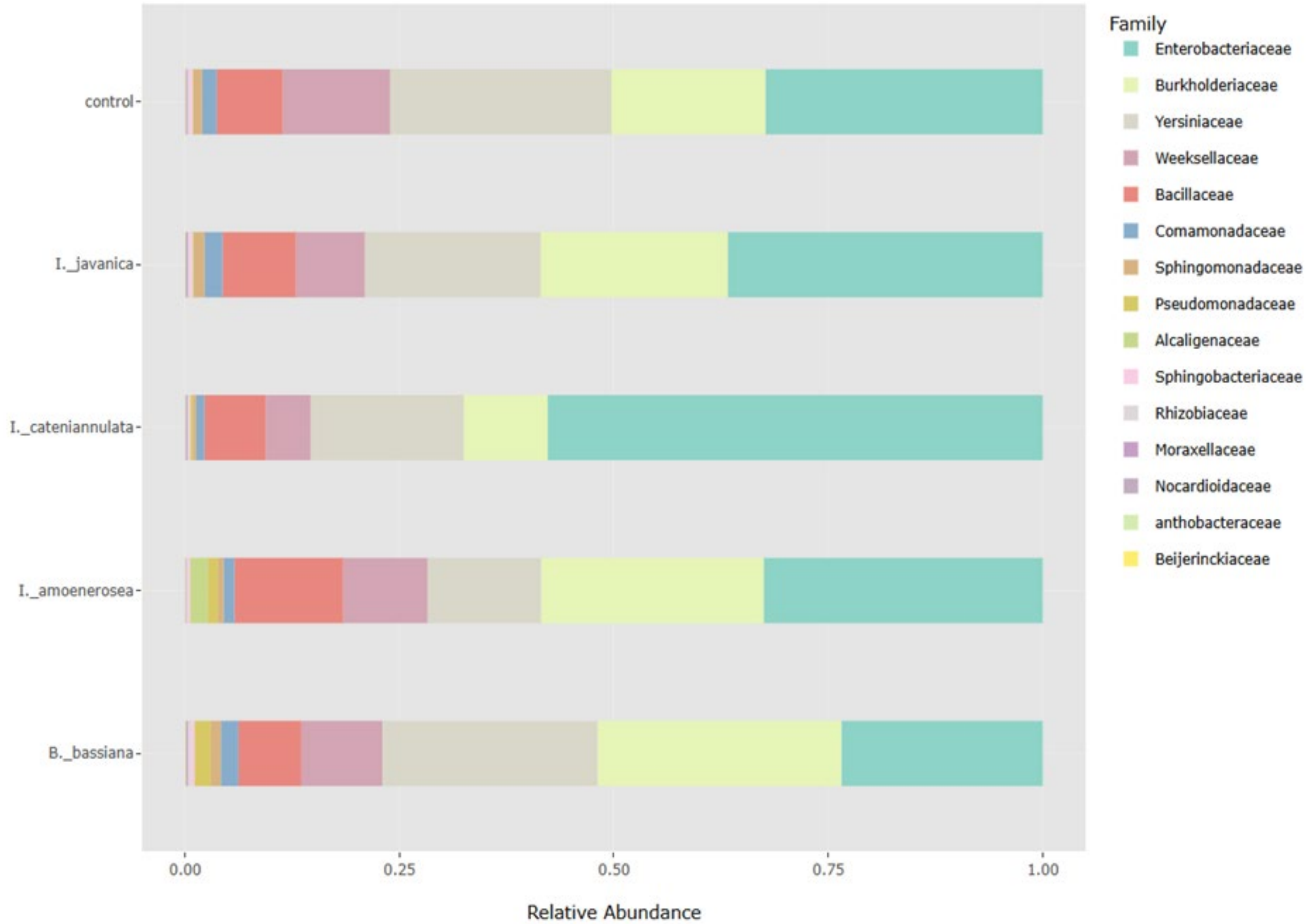

Supplementary Figure S2. Relative abundance of fifteen major bacterial families following fungal infection.

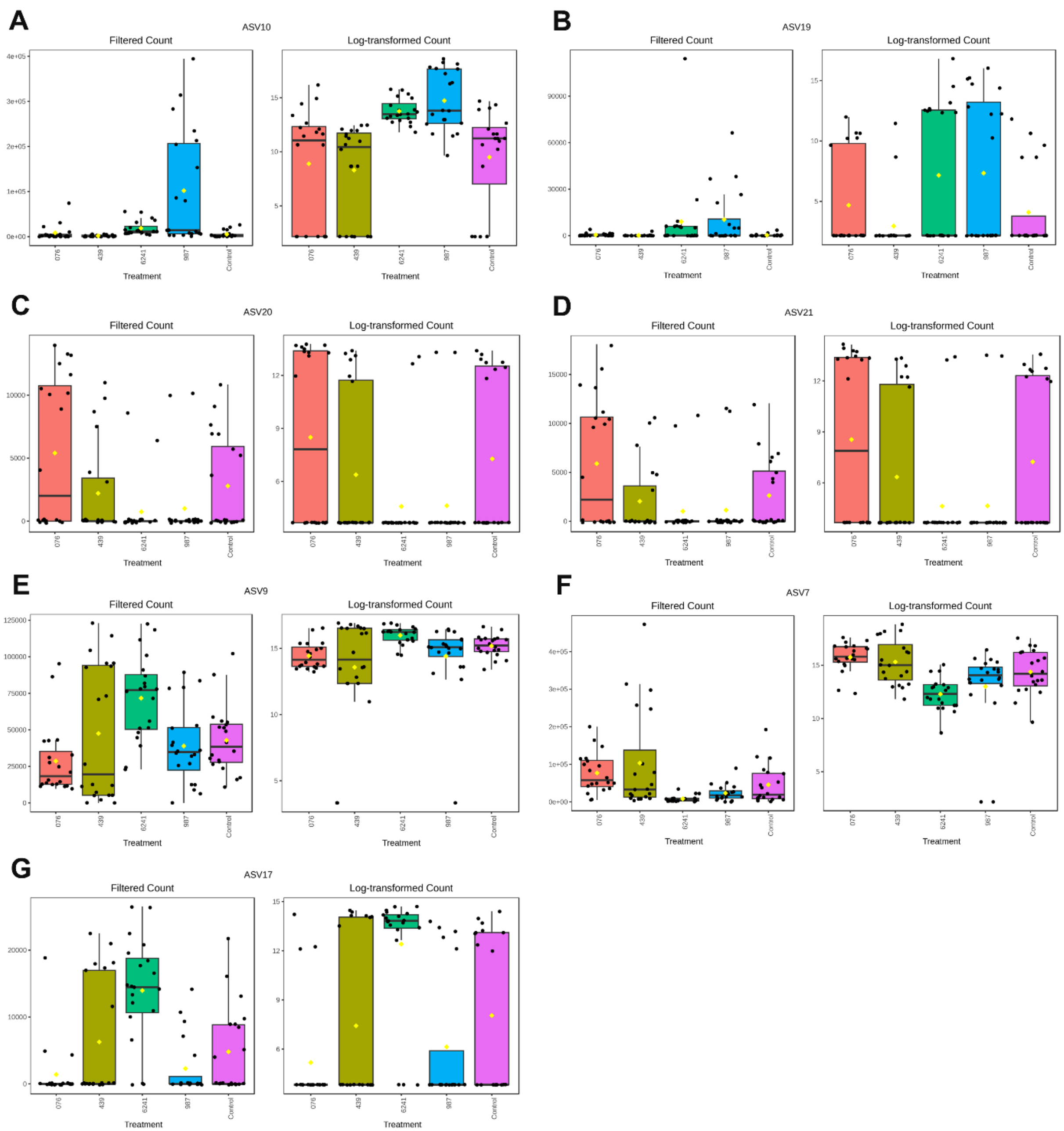

Supplementary Figure S3. Bar charts depicting the significant differential abundance of ASVs between infection groups and control. Data analyzed through Linear Discriminant Analysis Effect Size (LEfSe).

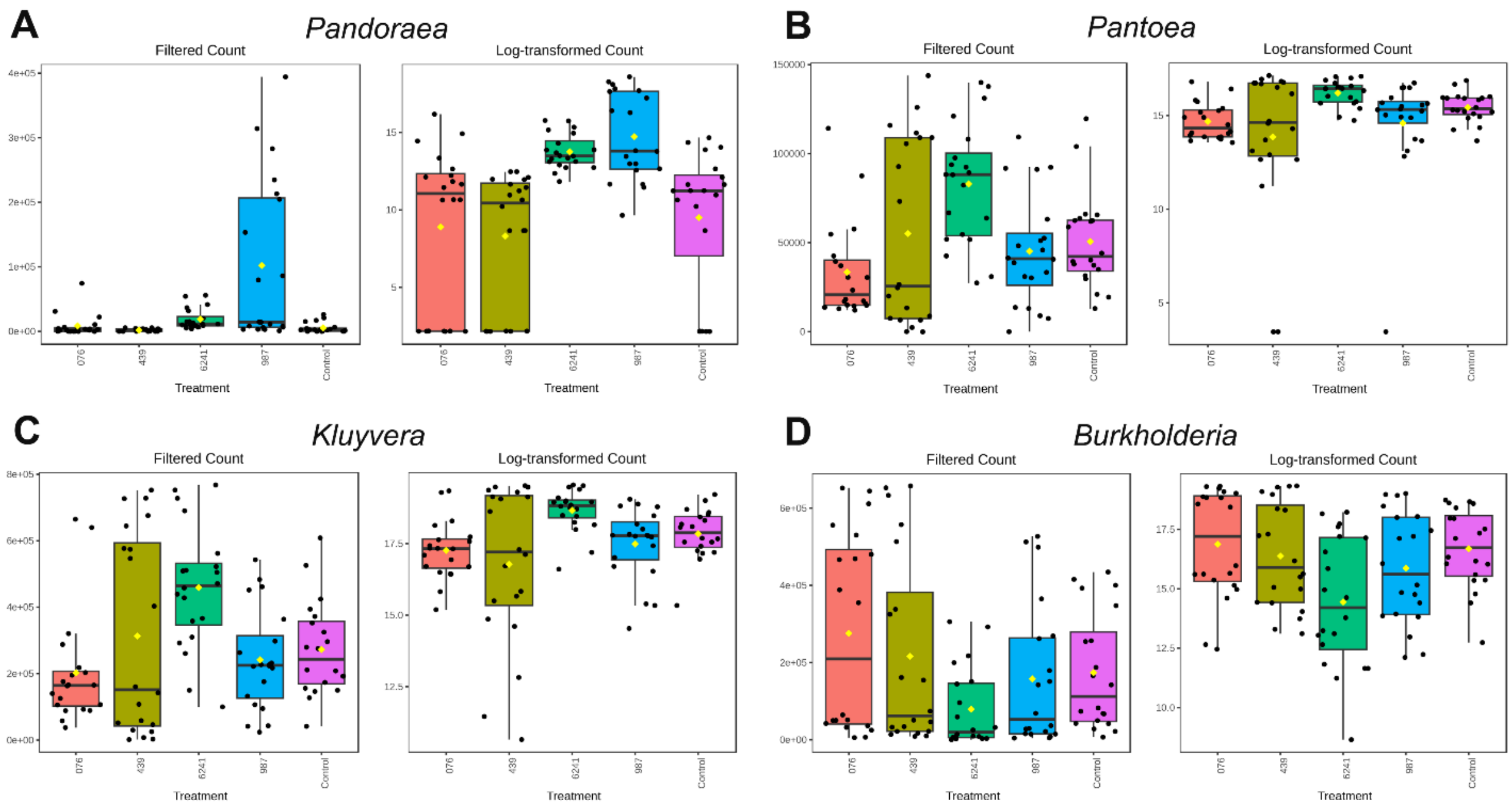

Supplementary Figure S4. Bar charts depicting the significant differential abundance at the genera level associated with fungal infection. Data analyzed through Linear Discriminant Analysis Effect Size (LEfSe).
